# Amino Acid Variants at the P94 Position in *Staphylococcus aureus* Class a Sortase Modulate Substrate Binding and Enzyme Activity

**DOI:** 10.1021/acs.biochem.6c00099

**Published:** 2026-04-02

**Authors:** Noah Cox-Tigre, Mia E. Stewart, Jackson Tucker, Erich G. Walkenhauer, Cooper S. Wilce, John M. Antos, Jeanine F. Amacher

**Affiliations:** Department of Chemistry, 1632 Western Washington University, Bellingham, Washington 98225-9008, United States

## Abstract

The surface of Gram-positive bacteria is a highly regulated environment with specific attachment of proteins required for viability. Sortase enzymes recognize and ligate substrates to the peptidoglycan layer in these microorganisms, which can be highly pathogenic (e.g., *Staphylococcus aureus*). As such, sortases represent a potentially novel target for antibiotic development. In addition, the catalytic activity of sortase enzymes is utilized in sortase-mediated ligation (SML) engineering approaches for a variety of uses. In SML experiments, engineered variants of *S. aureus* sortase A (saSrtA) are the most widely used enzymes. Structural analyses of experimental saSrtA structures revealed that the P94 position interacts directly with Y187 when saSrtA is in its inactive conformation. Interestingly, P94 is mutated in the previously engineered pentamutant (or saSrtA5M), to P94R. We wanted to interrogate single mutations at P94 further to characterize its effect on activity and/or substrate specificity. We created 18 P94X mutations (except cysteine) and tested relative activity for 4 substrate sequences: LPATG, LPETG, LPKTG, and LPSTG. We identified several P94 variants that both outperformed and contained differing specificity as compared to P94R. We tested P94A and P94D saSrtA5M variants and found that, depending on the substrate, these variants could outperform saSrtA5M in activity >3-fold. Finally, we compared saSrtA5M and P94D saSrtA5M in a model sortase-mediated ligation reaction using a LPKTG substrate and saw ∼2-fold greater product formation. We argue that future studies which combine rational design and high throughput approaches, e.g., directed evolution, may result in sortase variants with increased SML potential.

## Graphical abstract

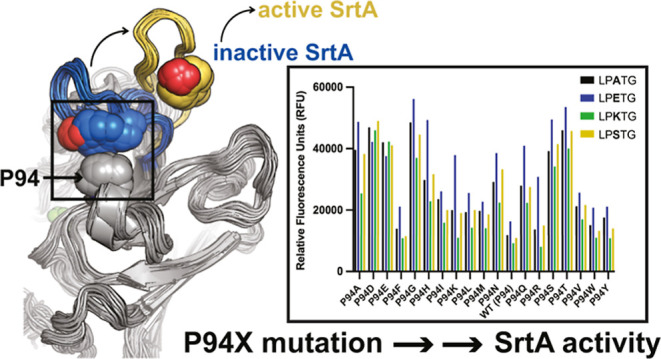

## Introduction

The thick peptidoglycan layer that defines Gram-positive bacteria, which include pathogens such as *Staphylococcus aureus*, *Streptococcus pyogenes*, and *Enterococcus faecalis*, provides a protective layer for these organisms.^1^ This surface is a dynamic environment, and acts as a scaffold for proteins that are critical for viability, defense, pathogenesis, and cellular function.^1^ Sortase enzymes, specifically class A sortases (SrtAs), are cysteine transpeptidases localized to the cleavage furrow of dividing Gram-positive bacteria, and which covalently attach substrate proteins to the developing peptidoglycan layer.^1,2^ SrtA enzymes were first described in 1999.^3–5^ Even in those first studies, SrtA enzymes were identified as a potential novel target for antibiotic development, work that remains ongoing.^5–19^ In addition to interest in sortase enzymes as antimicrobial targets, sortase-mediated ligation (SML) strategies quickly emerged as a powerful protein engineering approach for *in vitro* and *in vivo* applications, including in nanobody/antibody drug conjugation, developing novel insulin derivatives, as both diagnostic and therapeutic tools for neurodegenerative disease, and in creating multivalent vaccines for SARS-CoV-2, among many other uses.^20–29^

The sortase catalytic mechanism is well understood. A core triad of His-Cys-Arg residues (for *S. aureus* SrtA, H120–C184-R197) facilitate the processing of the cell wall sorting signal (CWSS) sequence, LPXTG, where X = any amino acid (L = P4, P = P3, X = P2, T = P1, G = P1′), on a target protein.^30,31^ Recent work from ourselves and others argues that while R197 interacts with and stabilizes the bound LPXTG ligand, it is not directly responsible for catalysis.^26,32–34^ Instead, upon nucleophilic attack of the side-chain thiol of C184 on the carbonyl carbon of P1 Thr, the resulting oxyanion tetrahedral intermediated is stabilized by a conserved Thr immediately preceding the catalytic Cys (in *S. aureus* SrtA, T183) and the backbone amide of the amino acid immediately following the catalytic His (in saSrtA, T121).^26,33^ Overall, sortase catalysis follows a ping-pong mechanism, first involving the formation of a thioester-linked acyl enzyme intermediate. Transpeptidation is then completed when a second nucleophilic substrate, typically an N-terminal Gly, attacks the acyl-enzyme intermediate, resulting in covalent attachment of the two substrates via a new peptide bond.^26,31^

Despite the identification of over 10,000 enzymes in the sortase superfamily, SML experiments most commonly utilize engineered variants of the first SrtA discovered, *S. aureus* SrtA (saSrtA).^35,36^ Biochemical and structural characterization of a number of SrtA enzymes previously revealed both shared and unique attributes of saSrtA and SrtA enzymes from other organisms. For example, the catalytic domains of SrtA enzymes studied to date share relatively low catalytic efficiencies as compared to other enzymes.^31^ Uniquely, saSrtA and other SrtA enzymes in the *Staphylococcus* genera contain a calcium-binding site, an allosteric activator which initiates a conformational change and is required for full activation of the enzyme.^30,31,36^ Of several SrtA enzymes studied, saSrtA is the most specific for the canonical pentapeptide recognition motif, LPXTG.^37,38^ Finally, flexibility in a structurally conserved loop near the active site between the β7 and β8 strands (the β7–β8 loop) varies among SrtA enzymes. For example, in saSrtA, this loop shifts between two conformations in the apo and ligand-bound structures, PDB IDs 1IJA and 2KID (Figure 1A).^39,40^ In *Bacillus anthracis* SrtA (baSrtA), the β7–β8 loop undergoes a disordered-to-ordered transition upon ligand binding, PDB IDs 2RUI and 2KW8 (Figure 1A).^41,42^ In *S. pyogenes* SrtA (spySrtA), the conformation of the loop is similar in both apo and ligand-bound structures, PDB IDs 3FN5 and 7S51 (Figure 1A).^33,43^ The functional relevance of these differing SrtA characteristics is not well understood.

**Figure 1:**
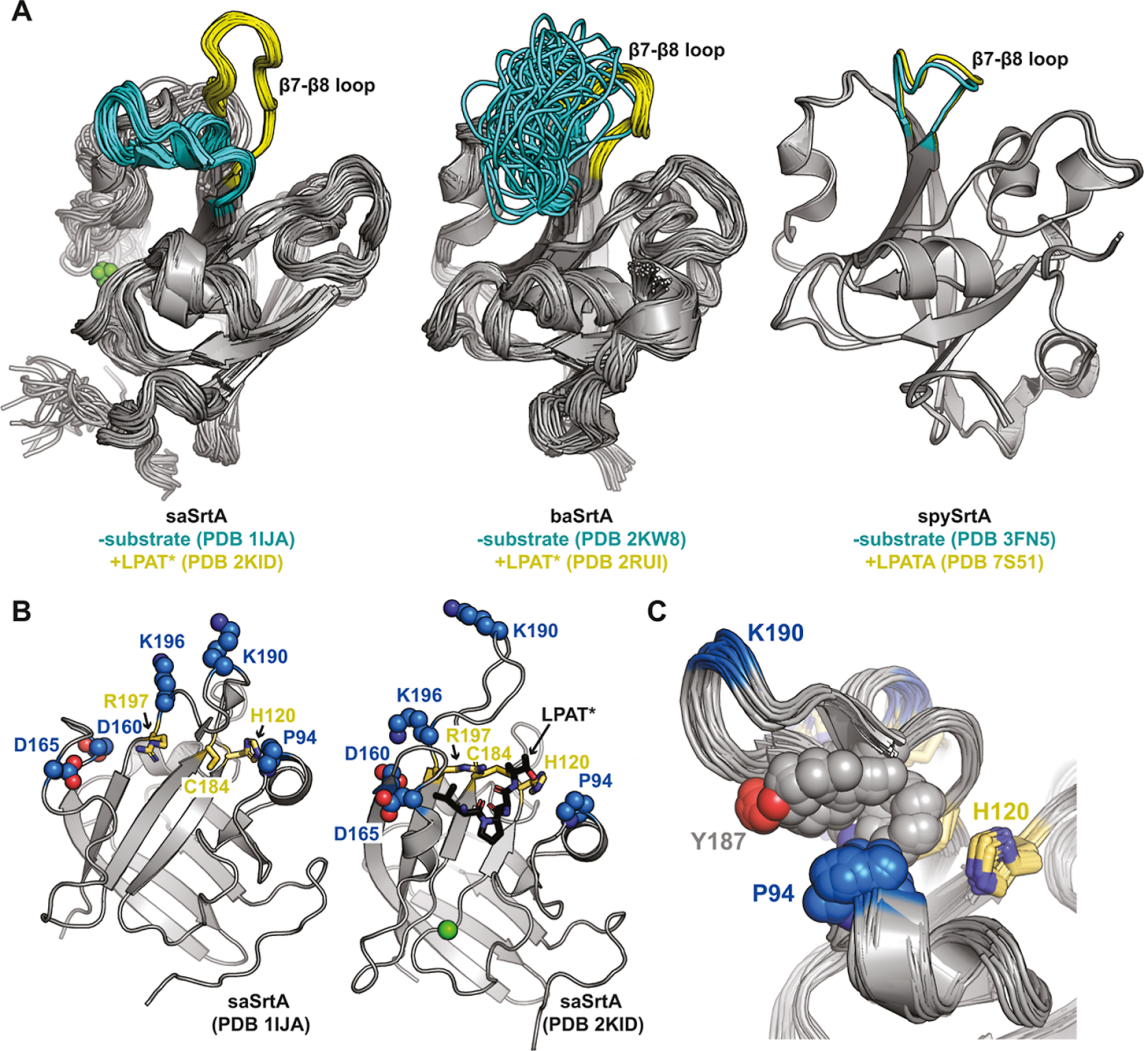
Experimental structures highlight differences in β7–β8 loop conformations without substrate bound, and an interaction between P94 and Y187 in *S. aureus* SrtA (saSrtA). (A) Experimental structures of *S. aureus* SrtA (saSrtA, PDB IDs 1IJA and 2KID),^39,40^ *B. anthracis* SrtA (baSrtA, PDB IDs 2KW8 and 2RUI),^41^ and *S. pyogenes* SrtA (spySrtA, PDB IDs 3FN5 and 7S51)^33,43^ are shown in cartoon representation with the β7-β8 loops colored cyan in the -substrate and yellow in the + substrate conformations. The calcium (Ca^2+^) ion is shown as a green sphere for saSrtA, which is allosterically activated by calcium. The saSrtA and baSrtA structures were determined using NMR and all available states are shown. (B) Mutated positions in the saSrtA5M pentamutant are highlighted as spheres and colored by heteroatom (C = blue, O = red, *N* = blue). The catalytic residues (H120, C184, R197) are shown as sticks and colored by heteroatom (C = yellow, *N* = blue, S = gold). The LPAT* peptidomimetic is shown for PDB ID 2KID as black sticks and colored by heteroatom. Ca^2+^ is shown as a green sphere. (C) All available NMR states are shown for saSrtA in its inactive state (PDB ID 1IJA), with the catalytic residues (H120, C184, R197) as yellow sticks and P94 and Y187 side chains as spheres. All highlighted residues are colored by heteroatom (C = yellow for catalytic residues, blue for P94, and gray for Y187, O = red, *N* = blue).

Directed evolution experiments previously identified a pentamutant (P94R/D160N/D165A/K190E/K196T) of saSrtA with >120-fold increased catalytic efficiency over wild type (WT).^44^ Subsequent work added two additional mutations (either E105K/E108A or E105K/E108Q), creating a heptamutant.^45,46^ Although the heptamutant (saSrtA7M) is approximately 3-fold less efficient than the pentamutant (saSrtA5M), it remains more active than the WT enzyme and does not require the Ca^2+^ cofactor; therefore, it is widely used in SML methodologies.^46,47^ As discussed in the original saSrtA5M work, the dramatic increase in catalytic efficiency is largely due to a substantial decrease in *K*_m_ with respect to recognition of the LPETG peptide substrate.^44^ Chen et al. reasoned that this is likely influenced by the location of the mutated residues and the resulting effects on the conformation of the structurally conserved loops near the ligand binding groove, like the β7–β8 loop.^44^ Consistent with this, the K190E and K196T mutations are located within the β7–β8 loop (Figure 1B). Furthermore, D160N and D165A are within the β6–β7 loop, which undergoes a disordered-to-ordered transition upon calcium binding and was previously shown to affect specificity in the P4 substrate position (Figure 1B).^44,48–50^ While P94 is adjacent to the ligand binding groove, it is not located in either of these loops, although of the 5 mutated sites, it is in closest proximity to the ligand itself (Figure 1B).

Considering these observations, we wanted to better understand the saSrtA conformational transition between apo and ligand-bound structures, PDB IDs 1IJA and 2KID (Figure 1A). We observed that the side chain of P94 appears to make stabilizing hydrophobic interactions with Y187 and W194, both in the β7–β8 loop, only in the ligand-free conformation (Figure 1C). When mutated to P94R, we reasoned that these interactions would be disrupted, leading to an active conformation that mimics the ligand-bound form of the enzyme, wherein the β7–β8 loop is in the “open”, upward orientation (Figure 1A). However, we also observed that E189 in the β7–β8 loop is potentially positioned to positively interact with the Arg in the P94R saSrtA variant, which we hypothesize could favor the inactive, loop “down” conformation. Conversely, the LPETG peptide substrate in the directed evolution experiment contained a P2 Glu that could be beneficial to the P94R saSrtA variant by introducing an additional enzyme–substrate interaction. Notably, P94S was reported in early rounds of the directed evolution engineering experiment, and the stereochemical consequence of Ser versus the wild-type Pro is not known.^44^ To answer these questions and better understand the effect of mutation at the P94 position, we created 18 P94X mutations, excluding only P94C, which we reasoned may form a disulfide bond with C184. We tested the relative activity of all P94X variants and WT saSrtA with four substrate sequences that differed at the P2 position: LPATG, LPETG, LPKTG, and LPSTG. We found several P94X variants that outperformed the single P94R-containing mutant. When we replaced this mutation in saSrtA5M with either P94A or P94D, we saw enhanced activity for certain LPXTG sequences. Consistent with this, sortase-mediated ligation experiments showed ∼2-fold greater product formation for P94D saSrtA5M as compared to saSrtA5M using a LPKTG substrate. As previously shown in the development of saSrtA5M, enzyme kinetics assays confirmed that these results are largely driven by a decrease in *K*_m_. Taken together, our results suggest that a thorough understanding of structure-function relationships in sortase enzymes can be used to combine rational design with high throughput techniques to develop improved tools for the broad SML protein engineering field.

## Materials and Methods

### Protein Expression and Purification

Recombinant sortase variant expression and purification was performed as previously described.^33,36,51–53^ The WT saSrtA sequence used was UniProt ID SRTA_STAA8, catalytic domain residues 60–206. Prior to the protein sequence, we included a 6×-His-tag and TEV protease cleavage site (sequence MESSHHHHHHENLYFQS), as in our previous work.^53^ Briefly, pET28a­(+) plasmids (GenScript) for each variant were transformed into *Escherichia coli* BL21 (DE3) competent cells. One-liter growths using LB media and kanamycin (50 μg/mL) were grown at 37 °C to an optical density of 0.6–0.8, followed by induction with 1 mM isopropyl β-D-thiogalactoside (IPTG). The temperature was shifted to 18 °C for 18–20 h. Cell pellets were harvested via centrifugation at 6500 rpm over 10 min. Cells were lysed with SrtA lysis buffer (0.05 M Tris pH 7.5, 0.15 M NaCl, 0.5 mM EDTA) and the lysate was sonicated for 1 min in a 50 W, 50% duty cycle. The lysate was centrifuged for 30 min at 17,500 rpm. The supernatant was filtered and loaded onto a Cytiva HisTrap HP (5 mL) column. The column was washed in SrtA wash buffer (0.05 M Tris pH 7.5, 0.15 M NaCl, 0.02 M imidazole, 1 mM tris­(2-carboxyethyl)­phosphine hydrochloride (TCEP)), followed by elution with SrtA elution buffer (0.05 M Tris pH 7.5, 0.15 M NaCl, 0.3 M imidazole, 1 mM TCEP) using a linear gradient over 20 column volumes (CV). Purified protein was concentrated using a Millipore Amicon Ultra Centrifugal Filter (10 kDa filter). In this work, we did not add TEV protease to our purified proteins, which we previously showed does not affect saSrtA activity.^51^ The concentrated protein was further purified via size exclusion chromatography (SEC) on a Cytiva HiLoad 16/600 Superdex 75 pg column. Protein aliquots were flash frozen with liquid nitrogen and stored at −80 °C. Protein identity was confirmed using SDS-PAGE and LC-ESI-MS (Table S1).

### Peptide Synthesis

Peptide substrates were synthesized using the Biotage Initiatior + Alstra synthesizer. Synthesized peptides included the sequences: Abz-LPXTGGK­(Dnp), with X = A, E, K, and S, Abz = 2-aminobenzoic acid, and Dnp = 2,4-dinitrophenyl. All peptides were synthesized on Fmoc Rink-Amide MBHA resin (Anaspec) on 0.1 mmol scale, resulting in peptide substrates with C-terminal primary amides (−NH_2_). Coupling reactions were performed in NMP using 5.0 mol equiv of each appropriately protected amino acid or building block, 4.9 mol equiv of HBTU, and 10.0 mol equiv of DIPEA with microwave heating for 5 min at 75 °C. Installation of the 2,4-dinitrophenyl chromophore and Abz fluorophore was achieved through coupling of commercially available Fmoc- *l* -Lys­(Dnp)–OH (ApexBio) or Boc-2-aminobenzoic acid (Chem-Impex International), respectively. Fmoc removal between coupling cycles was achieved using a solution of 20% piperidine in NMP. After syntheses were completed, the resin-bound peptides were washed with dichloromethane and diethyl ether. Peptides were then cleaved from the solid support using 10 mL of 95:2.5:2.5 TFA/TIPS/H_2_O for 3 h at room temperature. The cleaved peptide solutions were then concentrated using a rotary evaporator, and the remaining residue was precipitated from dry ice chilled diethyl ether. The resulting solid was collected by centrifugation and allowed to air-dry overnight. Once dried, the crude peptides were dissolved in a minimum volume of acetonitrile, diluted with ∼10 mL of water and lyophilized. The crude peptides were then purified using a Dionex Ultimate 3000 HPLC system equipped with a Phenomenex Luna 5 μm C18(2) 100 Å column (10 × 250 mm) [aqueous (95% water, 5% MeCN, 0.1% formic acid)/MeCN (0.1% formic acid) mobile phase at 4.0 mL/min, method: hold 10% MeCN 0.0–2.0 min, linear gradient of 10–90% MeCN 2.0–15.0 min, hold 90% MeCN 15.0–17.0 min, linear gradient of 90–10% MeCN 17.0–17.01 min, re-equilibrate at 10% MeCN 17.01–19.0 min)]. Purified peptide fractions were concentrated on a rotary evaporator, lyophilized, and stored at −20 °C. The identity and purity of all peptides was verified by LC-ESI-MS (Table S1) and RP-HPLC, respectively. For use in biochemical assays, peptides were dissolved in 100% DMSO at a final concentration of ∼2–5 mM and stored at −20 °C.

### LC-ESI-MS Characterization of Sortase Variants and Peptides

Mass spectrometry characterization was achieved using a Advion CMS expression^L^ mass spectrometer interfaced with a Dionex Ultimate 3000 HPLC system. For pure peptides and reactions involving peptide substrates, separations upstream of the mass spectrometer were achieved with a Phenomenex Kinetex 2.6 μm C18 100 Å column (100 × 2.1 mm) [aqueous (95% water, 5% MeCN, 0.1% formic acid)/MeCN (0.1% formic acid) mobile phase at 0.3 mL/min, method: hold 10% MeCN 0.0–1.0 min, linear gradient of 10–90% MeCN 1.0–7.0 min, hold 90% MeCN 7.0–9.0 min, linear gradient of 90–10% MeCN 9.0–9.1 min, re-equilibrate at 10% MeCN 9.1–13.4 min]. For sortase variants, separations upstream of the mass spectrometer were achieved with a Phenomenex Aeris 3.6 μm WIDEPORE C4 200 Å column (100 × 2.1 mm) [aqueous (95% water, 5% MeCN, 0.1% formic acid)/MeCN (0.1% formic acid) mobile phase at 0.3 mL/min, method: hold 10% MeCN 0.0–1.0 min, linear gradient of 10–90% MeCN 1.0–7.0 min, hold 90% MeCN 7.0–10.0 min, linear gradient of 90–10% MeCN 10.0–10.1 min, re-equilibrate at 10% MeCN 10.1–13.25 min]. Deconvolution of protein charge ladders was achieved using Advion Data Express (version: 6.9.43.1) software.

### Sortase Activity Assays

Enzymatic activity was tested using fluorescence assays with the BioTek Synergy H1 plate reader. The following concentrations were used, unless otherwise noted in the text: 1 μM enzyme, 0.05 mM peptide, 5 mM hydroxylamine (H_2_NOH). Assays were performed using sortase running buffer (0.05 M Tris pH 7.5, 0.15 M NaCl, 0.01 M CaCl_2_). Assays were run for 2 h at room temperature using an excitation wavelength of λ = 320 nm. Data was recorded at the emission wavelength, λ = 420 nm. Time-matched fluorescence of the peptide alone (negative control) was subtracted from each data point. All assays were run as independent replicates with *N* = 3 for each enzyme–substrate pair, and standard deviation errors were calculated. Calculations and graphing were done using Excel and GraphPad Prism.

To complement fluorescence assays, as well as to monitor product formation from sortase-mediated ligation reactions, select pairings of sortase enzyme and peptide substrates were also monitored by HPLC. This was achieved using a Dionex Ultimate 3000 HPLC system and a Phenomenex Kinetex 2.6 μm C18 100 Å column (100 × 2.1 mm) [aqueous (95% water, 5% MeCN, 0.1% formic acid)/MeCN (0.1% formic acid) mobile phase at 0.3 mL/min, method: hold 10% MeCN 0.0–1.0 min, linear gradient of 10–90% MeCN 1.0–7.0 min, hold 90% MeCN 7.0–9.0 min, linear gradient of 90–10% MeCN 9.0–9.1 min, re-equilibrate at 10% MeCN 9.1–13.4 min]. The following reagent concentrations were used: 0.1 μM enzyme, 0.05 mM peptide, and 5 mM hydroxylamine (H_2_NOH) or 5 mM diglycine (Gly–Gly) in sortase running buffer (0.05 M Tris pH 7.5, 0.15 M NaCl, 0.01 M CaCl_2_). Assays were run for 2 h at room temperature and monitored at 360 nm. The extent of product formation was estimated from peak areas for the unreacted peptide substrate (Abz-LPXTGGK­(Dnp)) and the C-terminal cleavage fragment (GGK­(Dnp)). The identity of all relevant peptide peaks was confirmed using LC-ESI-MS (Table S1), as described above.

### Enzyme Kinetics Assays

Reaction mixtures were prepared in a total volume of 200 μL and included the following buffer and nucleophile components: 0.05 M Tris pH 7.5, 0.15 M NaCl, 0.01 M CaCl_2_, 1 mM hydroxylamine (H_2_NOH), and a final DMSO concentration of 5% (v/v). For all assays, the enzyme concentration was 2.79 μM. The substrate used was Abz-LPETGGK­(Dnp), at variable concentrations, which included 10 μM, 25 μM, 50 μM, 100 μM, 250 μM, 500 μM, and 1 mM. Assays were run in triplicate. Reaction time points (10, 60, 120, and 180 s) were obtained by quenching aliquots of each reaction with glacial acetic acid (7:1 final ratio of reaction mixture to acetic acid). Samples were analyzed via HPLC using a Dionex Ultimate 3000 HPLC system and a Phenomenex Kinetex 2.6 μm C18 100 Å column (100 × 2.1 mm) [aqueous (95% water, 5% MeCN, 0.1% formic acid)/MeCN (0.1% formic acid) mobile phase at 0.3 mL/min, method: hold 10% MeCN 0.0–1.0 min, linear gradient of 10–90% MeCN 1.0–7.0 min, hold 90% MeCN 7.0–9.0 min, linear gradient of 90–10% MeCN 9.0–9.1 min, re-equilibrate at 10% MeCN 9.1–13.4 min]. Samples were monitored at 360 nm, and two major peaks were observed for the unreacted peptide substrate (Abz-LPETGGK­(Dnp)) and the C-terminal cleavage fragment (GGK­(Dnp)). The identity of these peaks was confirmed using LC-ESI-MS (as described above), and initial velocities (μM/s) were estimated from their corresponding peak areas. GraphPad Prism was used to fit the Michaelis–Menten equation, and extract *V*_max_ and *K*_m_ values for each replicate; *k*_cat_ was calculated using the equation *V*_max_ = *k*_cat_*­[Enzyme]_total_.

### Structural Modeling

The AlphaFold3 server was used for structural modeling.^54^ PyMOL was used for structural figures and analyses, including the APBS plug-in to calculate electrostatic surface potential maps.

## Results and Discussion

### Relative Activities of P94X saSrtA Variants with the LPETG Substrate

To assess the impact of variations in the P94 saSrtA position, we first expressed and purified 18 single mutations. We included all amino acids except for the P94C variant due to the proximity of this residue to the catalytic C184 position, with an average distance of 8.3 Å (minimum distance = 7.7 Å) between the Cα atoms of P94 and C184 over the 25 NMR states of PDB 1IJA. Although this is outside the range of 3.0–7.5 Å previously observed for disulfide bonded cysteines, we reasoned that this was close enough that potential disulfide bond formation may perturb overall folding of the enzyme.^55^ All variants were expressed and purified using similar protocols as described previously and as in the Materials and Methods (Table S1).^33,36,51–53^

To compare relative activities of our P94X saSrtA variants, we utilized a fluorescence resonance energy transfer (FRET)-based peptide cleavage assay. We labeled a LPXTG-containing peptide at the N-terminus with a 2-aminobenzoyl (Abz) fluorophore, and at the C-terminus with a 2,4-dinitrophenyl (Dnp) quencher, as previously described and as in the Materials and Methods.^33,36,37,51–53,56^ The general sequence of our synthesized peptide substrates was Abz-LPXTGGK­(Dnp), with X = Glu (E) here, as this was the substrate used in the directed evolution study that produced the saSrtA pentamutant (saSrtA5M).^44^ For clarity, we will omit the Abz- and GK­(Dnp) residues when referring to these substrates moving forward, e.g., LPETG for Abz-LPETGGK­(Dnp). Upon sortase-mediated cleavage between the P1/P1′, or T/G, positions, we observed an increase in Abz fluorescence. While this assay only monitors the first step of the reaction, recognition of the substrate and formation of the acyl-enzyme intermediate was previously shown to be the rate-limiting step of the overall sortase reaction.^57–59^ Furthermore, a previous kinetic analysis of P94S saSrtA revealed a 3-fold decrease in *K*_m_ for this mutant with no change in *k*_cat_ (WT: *K*_m_ = 7.6 mM, *k*_cat_ = 1.5 s^–1^; P94S: *K*_m_ = 2.5 mM, *k*_cat_ = 1.6 s^–1^, for the LPETG substrate), suggesting that variation at this residue affects this initial step of the reaction.^44^

In our first experiment, we tested 19 saSrtA enzymes, including our variants and WT, with the LPETG peptide (Figures 2A and S1). Initially, we used an enzyme concentration of 5 μM and the peptide substrate at 50 μM, consistent with our previous work.^33,36,37,51–53,56^ However, this proved to be too high of a concentration to capture the early time points for several of our variants, and we decreased our P94X saSrtA concentration to 1 μM, as described in the Materials and Methods. To directly compare relative activities with the WT enzyme, we decided to focus our analyses on a time point relatively early in our *t* = 2 h experiment, before we observed any fluorescence plateaus. Therefore, we chose to normalize relative fluorescence at *t* = 20 min as compared to the WT saSrtA enzyme (Figure 2B). Notably, our results indicated that only the P94F variant showed lower activity than the WT enzyme. Interestingly, while P94R (the mutation included in saSrtA5M) had 1.89-fold higher relative fluorescence at *t* = 20 min, as compared to WT, this was far from the highest activity variant. Indeed, several mutations resulted in >2-fold higher changes as compared to WT, including A (3.0-fold), D (2.6), E (2.3), G (3.4), H (2.9), K (2.1), N (2.4), Q (2.5), S (3.0), and T (3.3) (Figure 2B). Of these, P94G, P94S, and P94T showed ≥ 3-fold increases in relative fluorescence on average as compared to WT (Figure 2B). Of the additional positively charged amino acids, P94K (2.1-fold as compared to WT) behaved similarly to P94R, while P94H was relatively more active at *t* = 20 min (2.86-fold as compared to WT) (Figure 2B).

**Figure 2:**
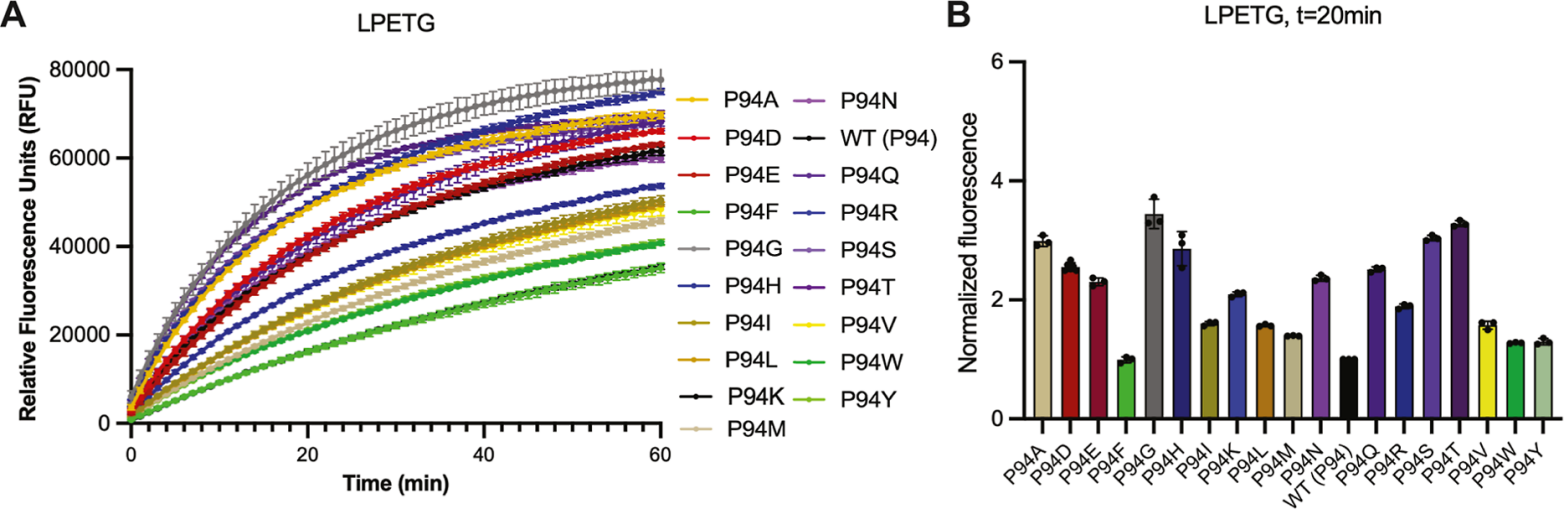
Activity assay data for P94X saSrtA variants and the LPETG substrate sequence. (A) Triplicate data (shown as averaged values with error bars equal to standard deviation) are shown for the P94X saSrtA variants during the first 60 min of the activity assay (total time = 2 h). Variants are colored as in the key and correspond to the colors used in (B). The full 2 h time course data is in Figure S1. Calculated initial velocities are in Figures S2–S3 and Table S2. (B) Normalized fluorescence (as averaged values ±standard deviation) for the P94X saSrtA variant data at the *t* = 20 min time point. Normalization is as compared to the WT data, which is equal to 1.

The P94 position is located on an α-helix between the β2 and β3 strands, and is situated almost directly across the peptide-binding cleft from the catalytic C184 residue (Figure 3A). Based on experimental structures, P94 forms a hydrophobic interaction with Y187 in the ligand-free state (PDB 1IJA), a position in the β7–β8 loop (Figure 1C).^39^ As discussed above, there is a conformational change in the β7–β8 loop upon substrate-binding that dramatically increases this distance (Figure 3B). In the NMR experimental structures, the loop “closed, down” inactive conformation revealed a distance of 7.3 ± 0.2 Å (PDB 1IJA), and the loop ‘up, open’ active conformation was 17.1 ± 0.3 Å (PDB 2KID) (Figures 1A, 3B). Therefore, we predict that a P94X mutation may affect overall flexibility and dynamics in the β7–β8 loop. Previous calcium titration NMR experiments using saSrtA confirmed that residues in the β7–β8 loop change conformation upon the addition of calcium, e.g., V193 and W194.^60^ This work primarily focused on the structure of the β6–β7 loop and similar experiments would be interesting in the presence of differing P94X variants to ask if relative flexibility differs upon mutation of this position.

**Figure 3:**
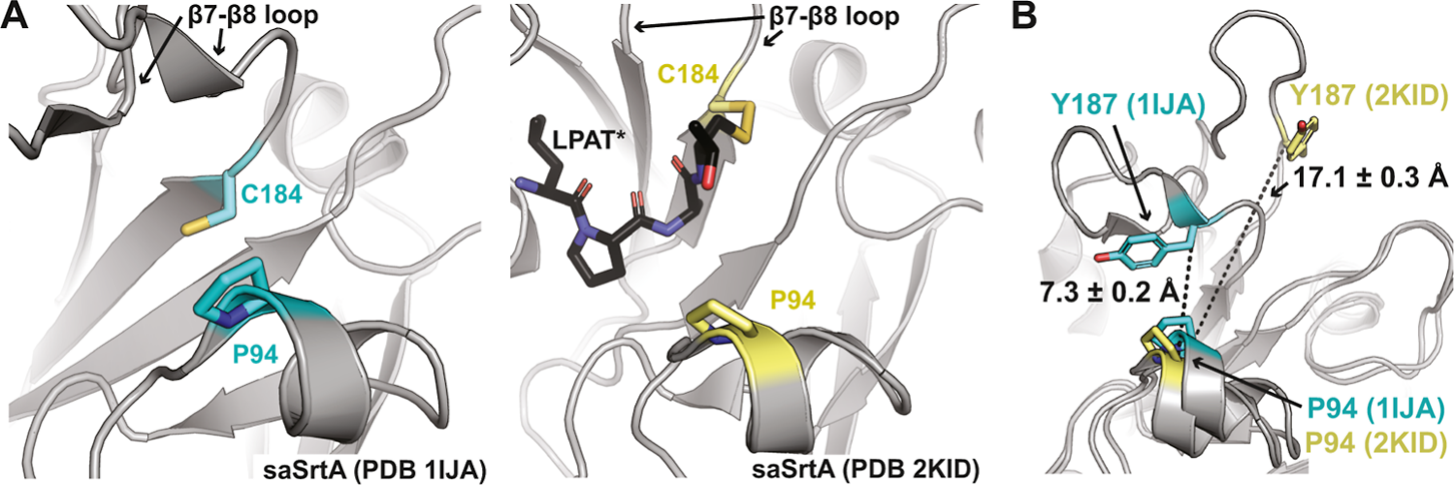
Distance between P94 and Y187 in saSrtA experimental structures. (A) The location of P94 with respect to the catalytic C184 residue (or Y187 in B) is highlighted in the WT saSrtA structures (PDB ID 1IJA (left) and 2KID (right).^39,40^ SaSrtA is in gray cartoon, and the side chain atoms of P94 and C184 are shown as sticks and colored by heteroatom (C = cyan for 1IJA and yellow for 2KID, *N* = blue, S = gold). The LPAT* peptidomimetic in PDB 2KID is in black sticks and colored by heteroatom (C = black, *N* = blue, O = red, S = gold). In (B), distances between Cα atoms are labeled and shown as black dashes.

### Relative Activities of P94X SaSrtA Variants with LPATG, LPKTG, and LPSTG

We next wanted to directly test the effect of P2 (LPXTG) identity on relative activity. The P94 amino acid is in a stereochemical environment such that we predicted it may be able to directly interact with the P2 (LPXTG) position of the substrate. Therefore, we wondered if there may be transient interactions during substrate binding/unbinding that could differentially affect the *k*_on_ and/or *k*_off_ for different substrate sequences. While this would be challenging to experimentally test due to the expected relatively high overall *K*_m_ for SrtA and peptide substrates (in the mM range), we reasoned that we could estimate the effects on these parameters by measuring relative activities. Thus, we tested our P94X variants with sequences that varied at the P2 position. In addition to LPETG, we chose to test P2 = A, K, and S due to the differing chemical properties of these amino acids. For example, while E is negative, K is positive, S is polar and uncharged, and A is small and hydrophobic.

Peptide substrates were synthesized as described above and as in the Materials and Methods, and activity assays were performed as with the LPETG substrate (Figure 2). Again, we focused our analyses on the *t* = 20 min time point (Figure 4A–C). However, we also calculated initial velocities (in RFU/min) for all P94X variants with all substrates, including LPETG (Figures S2–S3, Table S2). Our data revealed that while there were particular P94X variants that showed the highest relative activities for several of these substrates, specific P2 preferences also emerged (Figure 4A–C). For example, if we look at variants with an average increase in relative activity of >3-fold as compared to WT, they were (with shared P94X variants underlined): LPATG: A, D, E, G, S, and T; LPETG: G, S, and T; LPKTG: D, E, G, S, and T; and LPSTG: A, D, E, G, N, S, and T (Figures 2, 4).

**Figure 4:**
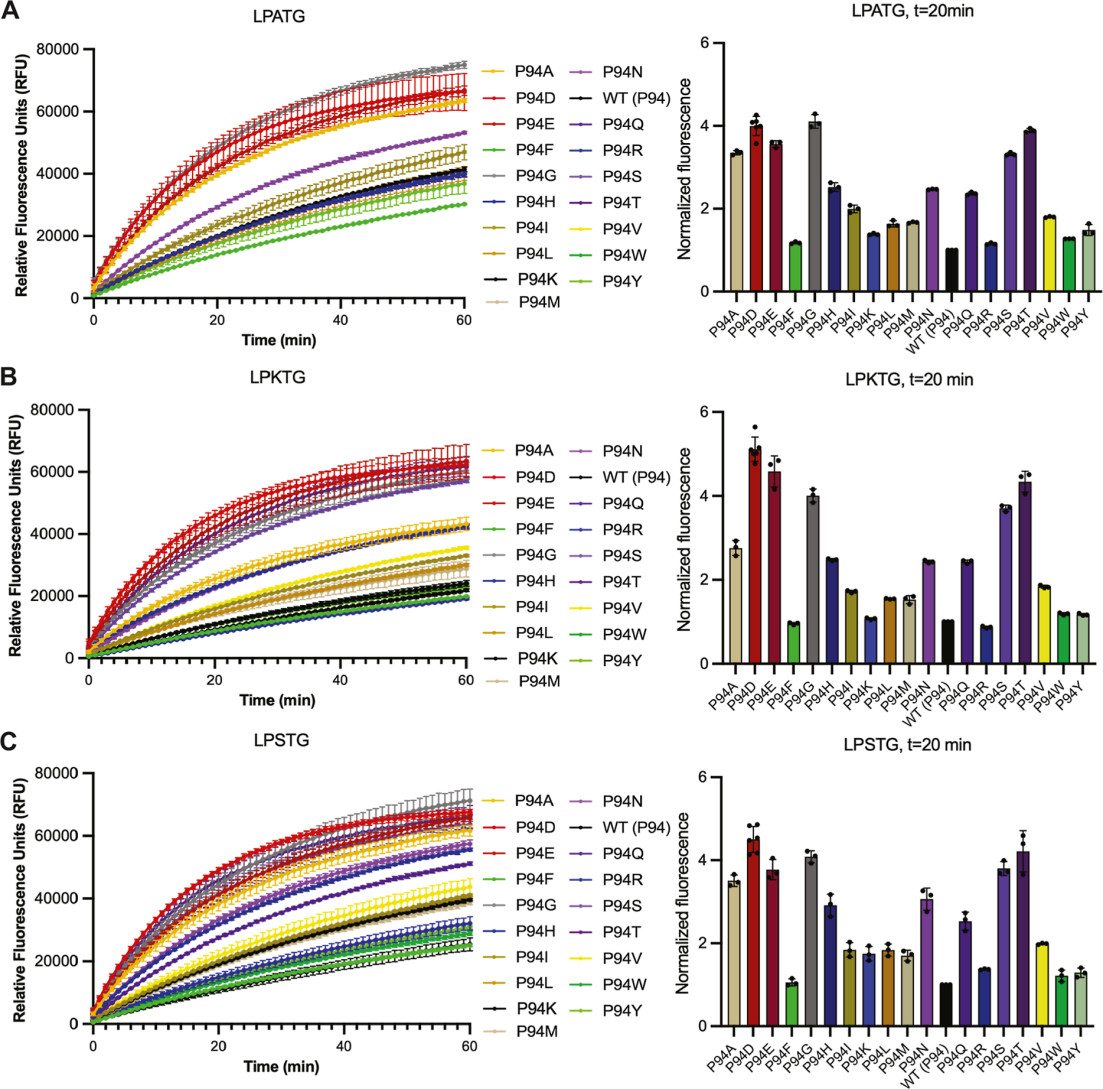
Activity assay data for P94X saSrtA variants and LPATG, LPKTG, and LPSTG substrate sequences. Triplicate data (shown as averaged values with error bars equal to standard deviation) are shown for the P94X saSrtA variants with LPATG (A), LPKTG (B), and LPSTG (C) peptide substrates during the first 60 min of the activity assay (total time = 2 h). Normalized fluorescence (as averaged values ±standard deviation) at the *t* = 20 min time point is also shown for each substrate (normalized to WT activity, which is equal to 1). Variants are colored as in the key, and corresponding to the colors used in Figure 2. The full 2 h time course data is in Figure S1. Calculated initial velocities are in Figures S2–S3 and Table S2.

To better investigate specific positive and/or negative preferences, we created a heat map of the average normalized fluorescence values for each P94X variant as compared to its relative fluorescence for LPETG at *t* = 20 min (Figure 5A). In general, the lowest relative activities are for LPKTG, which we reasoned may be due to the proximity of R197, and repulsive electrostatic interactions. We also analyzed specificity for P2 identify in the WT saSrtA enzyme (Figure 5B), and saw that at *t* = 20 min, WT saSrtA activity, as compared to LPETG, is 0.7× for LPATG, 0.6× for LPKTG, and 0.7× for LPSTG. Therefore, the WT enzyme prefers E > A > S > K at the P2 position (Figure 5B). Our results are largely consistent with previous data that investigated P2 specificity in saSrtA, which shows relatively minor variability in a ligation assay (*t* = 30 min) for P2 = A, E, and S (K was not tested).^61^ More dramatic differences from this previous work suggested positive preferences for P2 = M (>2-fold higher activity as compared to a P2 E) and negative preferences for P2 = G, I, T, and V (>2-fold lower activity as compared to a P2 E).^61^

**Figure 5:**
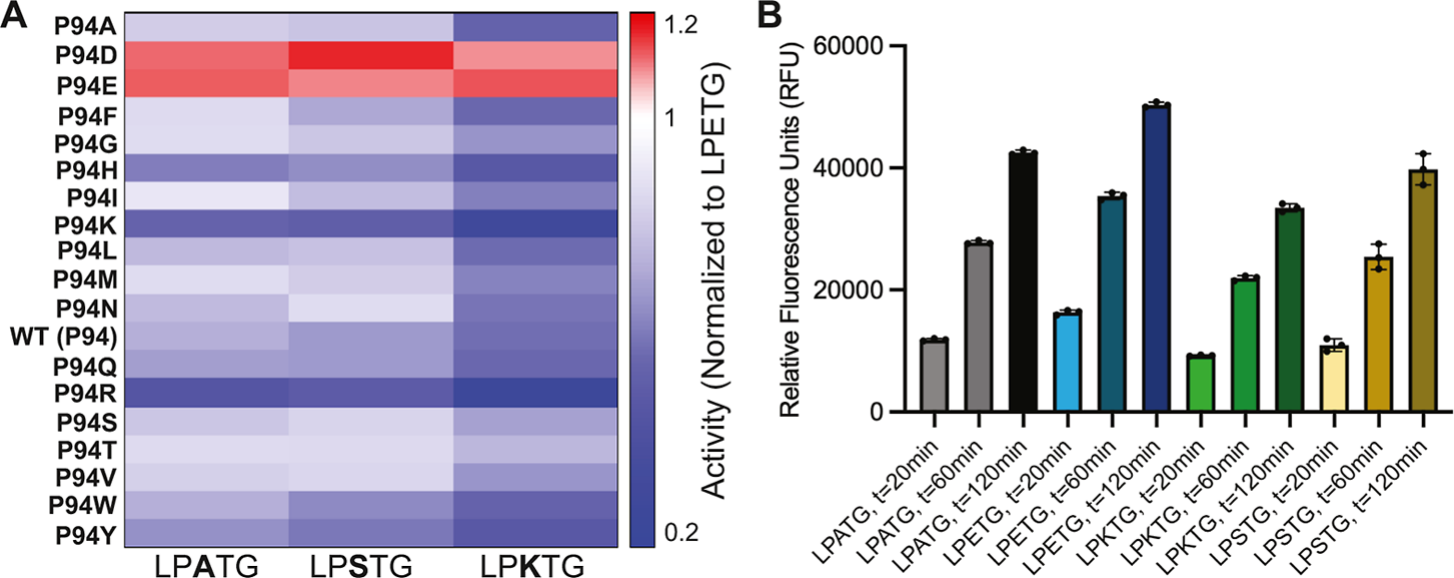
Relative P2 preferences for P94X saSrtA variants and WT. (A) A heat map comparing differences in relative fluorescence unit (RFU) values at *t* = 20 min for each P94X saSrtA variant, as compared to its value for the LPETG substrate (Figures 2,4). For example, the red colors for P94D and P94E saSrtA with the LPATG, LPKTG, and LPSTG substrates indicate that these are preferred over LPETG (RFU_LPXTG_/RFU_LPETG_ is > 1). (B) RFU values for WT saSrtA at *t* = 20,60,120 min as a function of the P2 position in the LPXTG substrates.

As shown in our heat map analyses, there were clear preferences that were not apparent when we normalized our fluorescence data to the WT enzyme (Figure 5A). Most were relatively minor and in general all P94X variants (except for P94D and P94E) disfavored LPKTG. There was an additional P2-specific effect observed in the P94R and P94K saSrtA variant data, where, as compared to LPETG, P94R average fluorescence was 0.4× for LPATG, 0.3× for LPKTG, and 0.5× for LPSTG (Figure 5A). We observed similar results when we graphed the fluorescence data for select individual P94X variants and WT and all substrates (Figure 6), as well as the average relative fluorescence values for each variant/peptide pair at *t* = 20 min (Figure 7). In general, all variants with only two exceptions (P94D and P94E) preferred the P2 Glu (LPETG), followed by LPATG or LPSTG (values within 85% of each other), then LPKTG (Figure 7). For P94D and P94E saSrtA enzymes, there was little difference in activity between substrates (Figures 6 and 7), although the P2 Glu was disfavored by both according to our heat map using *t* = 20 min data (Figure 5A).

**Figure 6:**
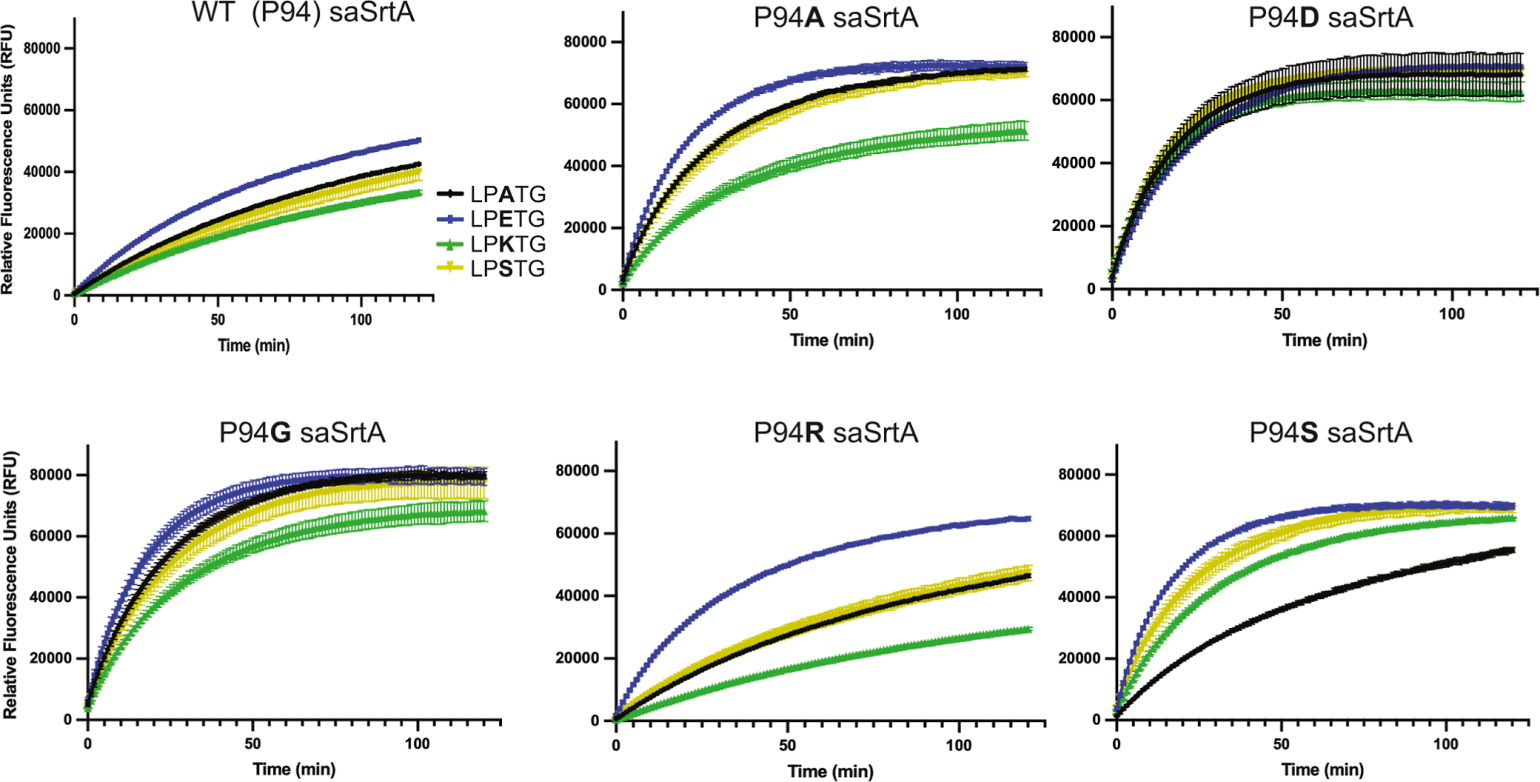
Activity assays for select variants as a function of P2 position in LPXTG substrates. Activity assays (*t* = 120 min) for select WT and P94X saSrtA variants as a function of the P2 position in the LPXTG substrates. Curves are colored as in the key to the right of the WT saSrtA data.

**Figure 7:**
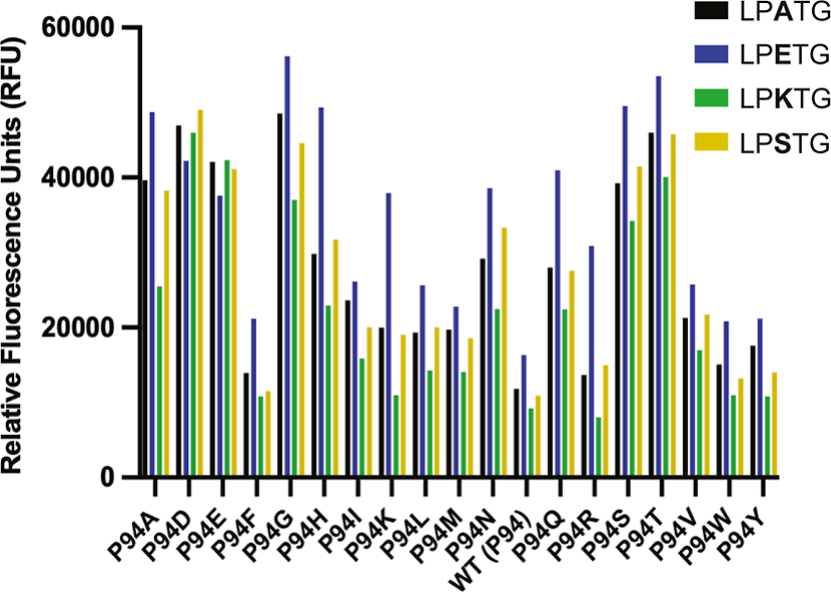
Relative fluorescence values for all P94X saSrtA assays at *t* = 20 min. Averaged RFU values for all P94X variants with all LPXTG substrates at *t* = 20 min. All experiments were performed in triplicate.

We modeled WT, P94D, and P94R saSrtA with Ca^2+^ and a QALPETGG peptide sequence that was previously studied, and which revealed similar activities for QALPETG as compared to an Ac-LPETG substrate control, using AlphaFold3.^54,60,62^ All models were well predicted (for WT: ipTM = 0.62, pTM = 0.87; for P94D: ipTM = 0.57, pTM = 0.88; for P94R: ipTM = 0.6, pTM = 0.89), and all output models aligned with overall RMSD values < 0.2 Å (Figure S4A,B). The WT saSrtA-QALPETGG structure aligned to the saSrtA-LPAT* NMR structure (PDB ID 2KID) with RMSD = 1.228 Å (over 1015 atoms) (Figure S4B). The electrostatic potential surface maps of the modeled saSrtA variants revealed that the relative surface charge of the catalytic R197 position appeared to vary with the different P94X identity (Figure 8). This was also true for the recently reported saSrtA5M with Ca^2+^ crystal structure (PDB ID 9WYA, Figure 8).^63^ The P94R saSrtA-QALPETGG-Ca^2+^ structure aligned to saSrtA5M with RMSD = 0.360 Å over 842 atoms (Figure S4C). Our previous work revealed that R197 (R216 in *S. pyogenes* SrtA, PDB ID 7S51) stabilizes the backbone of the substrate via hydrogen bonds.^33^ This may explain why the repulsive negative preference of P94D and P94E saSrtA for the P2 Glu is less pronounced than that for P94R and a P2 Lys, because in the P94D/E variants, there is the additional favorable interaction between the P2 Glu and the positively charged R197 amino acid. These models also suggest why the catalytic efficiencies, specifically *K*_m_, may vary with P94X mutation; here, the size and relative charge at the P94X position, which sits at the front of the substrate-binding cleft, may directly contribute to the relative *k*_on_ and *k*_off_ of binding (Figure 8). While experimental validation of these complexes is necessary, these models support the differences in activity data observed.

**Figure 8:**
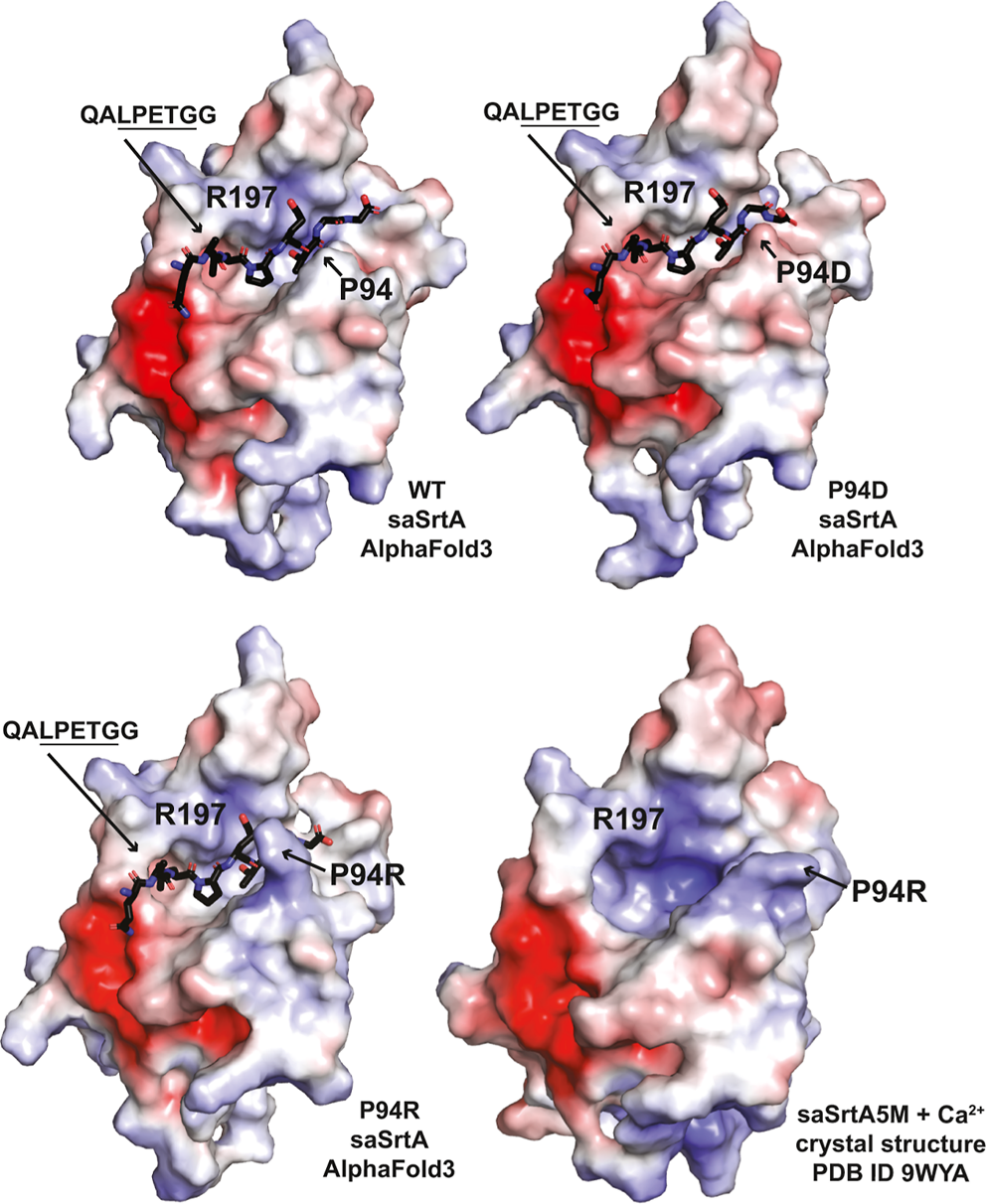
Electrostatic potential surface maps of WT, P94D, and P94R saSrtA-substrate-Ca^2+^AlphaFold3 models. Electrostatic potential surface maps (calculated using APBS in PyMOL) for AlphaFold3 models of WT saSrtA-QALPETGG, P94D saSrtA-QALPETGG, and P94R saSrtA-QALPETGG, as labeled. For comparison, the electrostatic potential map of the crystal structure of saSrtA5M (PDB ID 9WYA)^63^ is also shown. All models also included Ca^2+^. The scale is ±5 eV. The peptide substrate is shown as sticks and colored by heteroatom (C = black, O = red, N = blue). AlphaFold3 output data is in Figure S4.

### Effect of P94X Mutation on SaSrtA5M Activities

Based on our results, we hypothesized that the P94R mutation in saSrtA5M may exhibit different effects on activity for certain substrate sequences. We chose to test P94A and P94D in the context of the saSrtA5M enzyme, as these variants were relatively active compared to WT, and represent different chemical properties (Figures 2, 4A–C). We expressed and purified these variants as described in the Materials and Methods, and used our peptide cleavage assay to test their relative activities. We used enzyme concentrations of 1 μM and 0.1 μM. While the 1 μM concentrations allowed us to directly compare with our other assays, the 0.1 μM concentrations were required to capture initial activity.

Consistent with our data and previous work, the saSrtA5M variants were generally more active than all single P94X mutations at *t* = 20 min, except for saSrtA5M with LPKTG (Figure 9). Although the P94A and P94D saSrtA5M variants tested revealed similar activities for LPETG, this was not the case for the other P2 substrates. While the effect was most dramatic for LPKTG, as hypothesized due to the negative preference of P94R saSrtA for the P2 Lys, we found that P94A and P94D saSrtA5M also outperformed saSrtA5M (with the P94R mutation) at early time points for P2 = A, K, and S substrates (Figure 10). In our 1 μM assays, the relative fluorescence values were approximately equal by *t* = 60 min; however, this was not the case in our 0.1 μM assays at the *t* = 2 h time point (Figures 9 and 10). In these lower concentration assays, we again saw clear differences at the 20 min time point (Figure 10B). In addition, initial velocity measurements revealed even more dramatic effects, with initial average velocities for P94A saSrtA5M and P94D saSrtA5M as compared to saSrtA5M equal to 1.4× and 1.6× for LPATG, 1.1× and 0.9× for LPETG, 2.1× and 3.3× for LPKTG, and 1.4× and 1.5× for LPSTG, respectively (Figure 10C). Overall, this data suggests that P2 specificity plays a role in the widely used engineered saSrtA5M enzyme, and that different P94X mutations in the saSrtA5M context may be beneficial for substrates that are not LPETG.

**Figure 9:**
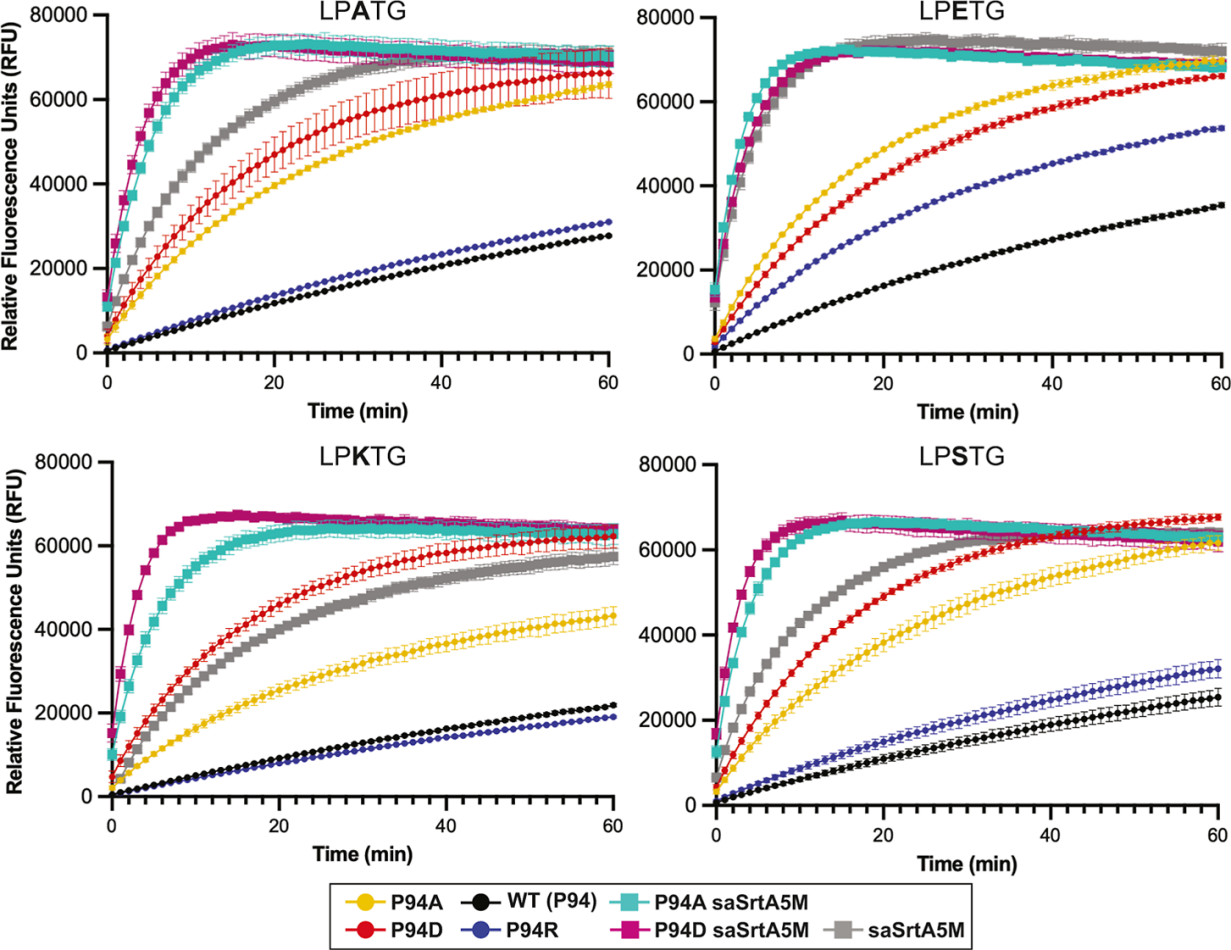
Activity assay data for select P94X saSrtA variants as compared to saSrtA5M variants. Triplicate data (shown as averaged values with error bars equal to standard deviation) are shown for select P94X saSrtA variants as compared to saSrtA5M, P94A saSrtA5M, and P94D saSrtA5M (key at bottom). Peptide substrates tested included: LPATG, LPETG, LPKTG, and LPSTG, as labeled. For all, the enzyme concentration used was 1 μM.

**Figure 10:**
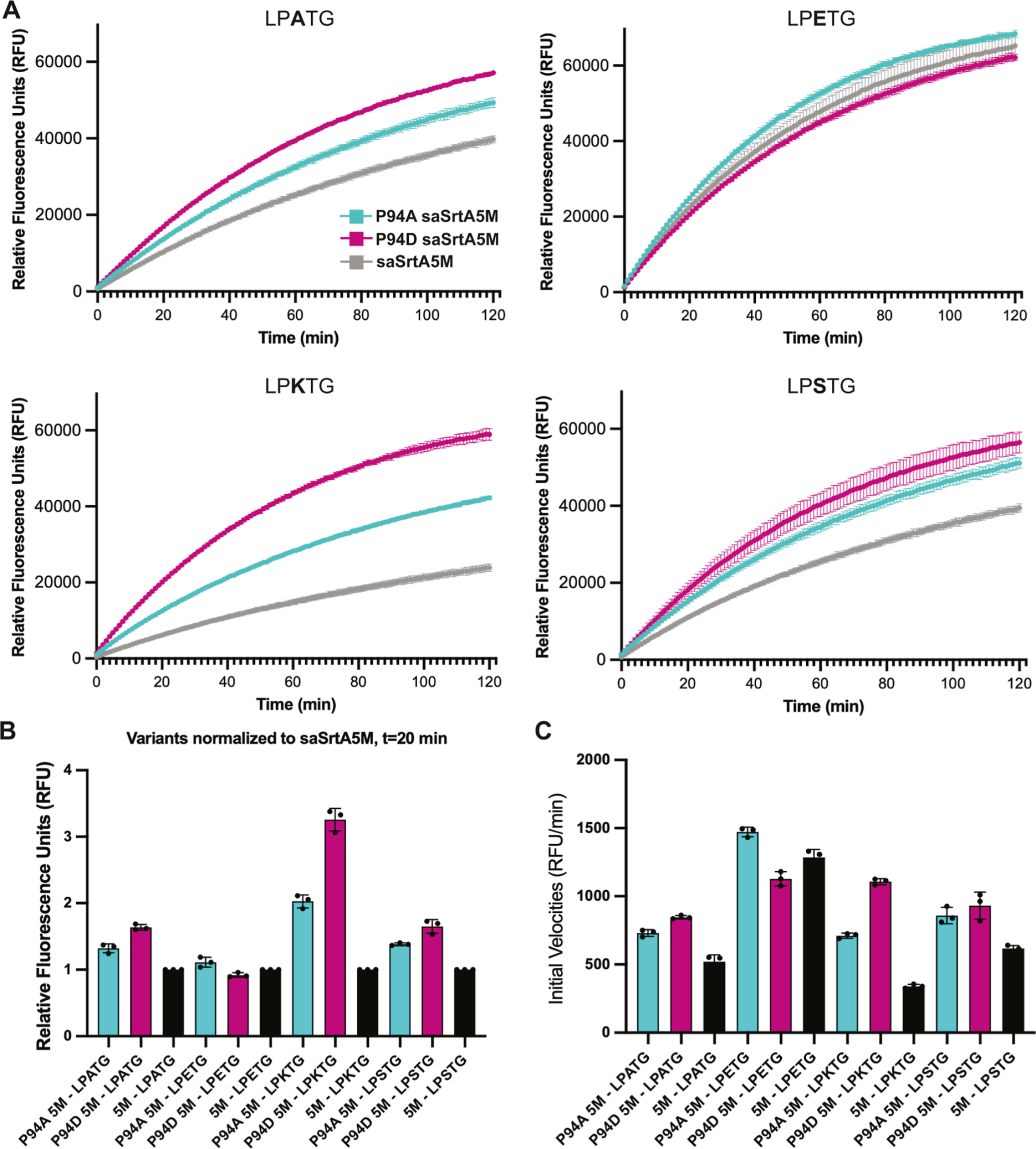
Effects of P94X mutation in the saSrtA5M pentamutant enzyme. (A) Triplicate data (shown as averaged values with error bars equal to standard deviation) for saSrtA5M, P94A saSrtA5M, and P94D saSrtA5M variants (key in top left). Peptide substrates tested included LPATG, LPETG, LPKTG, and LPSTG, as labeled. For all, the enzyme concentration used was 0.1 μM. (B) Relative fluorescence unit (RFU) values at *t* = 20 min, as normalized to the saSrtA5M (5M) data for each peptide substrate. Therefore, saSrtA5M = 1 for each of LPATG, LPETG, LPKTG, and LPSTG peptides. (C) Initial velocities (in RFU/min) for all saSrtA5M variants tested, with each peptide substrate, shown as average values ±standard deviation. Curves and calculated values are in Figure S2 and Table S2.

### Enzyme Kinetics and HPLC Cleavage Assays Using P94D SaSrtA Variants and SaSrtA5M

To further characterize the effects of P94X mutations, we next determined the kinetic parameters (*k*_cat_ and *K*_m_) of our P94D and P94R saSrtA and saSrtA5M variants and compared them to the standard WT saSrtA and saSrtA5M enzymes (Table 1). Initial reaction rates were calculated using an HPLC assay with fixed concentrations of enzyme and the H_2_NOH nucleophile, and various Abz-LPETGGK­(Dnp) substrate concentrations, as previously described and in the Materials and Methods (Figures 11a and S5).^64^ Importantly, our kinetics analyses were consistent with our peptide cleavage assay, as results were comparable to the relative fluorescence data described above. With respect to the literature, previously published *k*_cat_ and *K*_m_ values for sortase enzymes differ substantially, which we attribute to variations in the substrate peptide and nucleophiles used.^44,59,64–66^ Of these previous reports, the substrate/nucleophile pair most similar to our system is Abz-LPETGG-Dap­(DNP)-NH_2_ with a 2 mM pentaglycine (GGGGG) nucleophile, which resulted in *k*_cat_ = 1.10 ± 0.06 s^–1^ and *K*_m_ = 8.76 ± 0.78 mM for WT saSrtA and *k*_cat_/*K*_m_ = 125 ± 18 M^–1^ s^–1^ ^66^ While our values are lower than these (*k*_cat_ = 0.022 ± 0.001 s^–1^ and *K*_m_ = 0.185 ± 0.01 mM for WT saSrtA), they are shifted by a consistent factor of ∼50× (Table 1). As a result, our *k*_cat_/*K*_m_ = 120 ± 1 M^–1^ s^–1^ value strongly agrees with that previously reported.

**Table 1:** Kinetic Characterization of saSrtA Variants

	*k*_cat,_ s^–1^	*K*_m LPETG_, μM	*k*_cat_/*K*_m__LPETG_, M^–1^ s^–1^
**WT saSrtA**	0.022 ± 0.001	185 ± 10	120 ± 1
**P94D saSrtA**	0.020 ± 0.01	37 ± 3	550 ± 30
**P94R saSrtA**	0.017 ± 0.02	65 ± 5	280 ± 10
P94R/D160N/D165A/K190E/K190T (saSrtA5M)	0.04 ± 0.02	47 ± 11	770 ± 120
P94D/D160N/D165A/K190E/K190T (P94D saSrtA5M)	0.03 ± 0.01	20 ± 3	1500 ± 200

**Figure 11:**
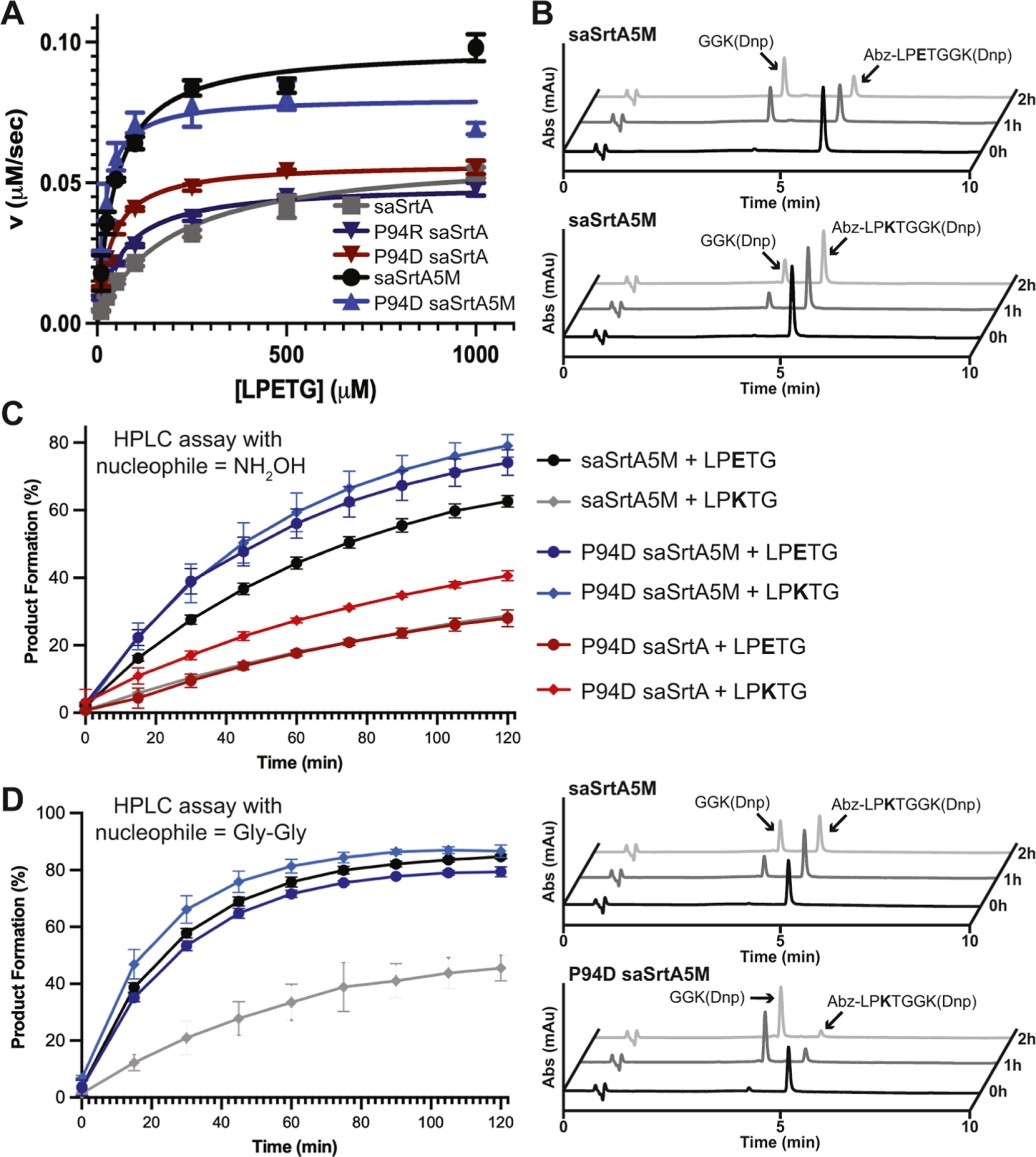
Enzyme kinetics and HPLC assays for saSrtA and saSrtA5M variants. (A) Average of replicate data (*N* = 3) for enzyme kinetics assays of saSrtA variants. Data was fit using the Michaelis–Menten equation in GraphPad Prism. Individual replicate data is in Figure S5. (B) Representative HPLC traces (360 nm) of sortase-mediated reactions utilizing a H_2_NOH nucleophile. Product formation catalyzed by the saSrtA5M enzyme was higher with the LPETG substrate, as compared to LPKTG, based on the areas of the substrate (Abz-LPXTGGK­(Dnp)) peak and cleavage product peak (GGK­(Dnp)) at indicated time points. (C) Quantification of sortase-mediated reactions (H_2_NOH nucleophile) using HPLC with standard deviation error bars shown. All experiments were conducted as technical triplicates and are described in the Materials and Methods. The extent of product formation was estimated from peak areas for the unreacted peptide substrate (Abz-LPXTGGK­(Dnp)) and the C-terminal cleavage fragment (GGK­(Dnp)). (D) Quantification (all) and representative HPLC traces for sortase-mediated ligation reactions (Gly–Gly nucleophile) using the substrates LPETG or LPKTG. The figure legend is the same as in (C), with saSrtA5M in black circles (+LPETG) and gray diamonds (+LPKTG), and P94D saSrtA5M in dark blue circles (+LPETG) and blue diamonds (+LPKTG). HPLC traces (360 nm) are rendered as in (B), highlighting the enhancement in reaction conversion for the LPKTG substrate when using the P94D saSrtA5M enzyme. Additional representative HPLC traces for saSrtA5M, P94D saSrtA5M, and P94D saSrtA catalyzed reactions are in Figure S6.

Our calculated kinetic parameters agreed with previously published values that the pentamutant mutations had a greater effect on *K*_m_ = 0.047 ± 0.011 mM (3.9-fold decrease as compared to WT) as compared to *k*_cat_ = 0.04 ± 0.02 s^–1^ (1.8-fold increase as compared to WT), although our results are not as dramatic as others reported.^44,64^ In general, we see a 6.4-fold increase in *k*_cat_/*K*_m_ for saSrtA5M (770 ± 120 M^–1^ s^–1^) as compared to WT saSrtA. Interestingly, while the *k*_cat_/*K*_m_ for P94R saSrtA is increased 2.3-fold as compared to WT (550 ± 30 M^–1^ s^–1^), the *k*_cat_/*K*_m_ for P94D saSrtA is increased 4.6-fold (Table 1). Consistent with this P94D versus P94R effect, we observed an additional 2-fold boost in *k*_cat_/*K*_m_ for P94D saSrtA5M (1500 ± 200 M^–1^ s^–1^) as compared to the saSrtA5M pentamutant (770 ± 120 M^–1^ s^–1^), which already contains the P94R mutation (Table 1). As noted above, these results were like our fluorescent peptide cleavage results (Figure 9); however, we were intrigued by the increased *k*_cat_/*K*_m_ for P94D saSrtA and P94D saSrtA5M versus P94R saSrtA and/or the pentamutant (with P94R) and wanted to further test these variants using additional HPLC-based assays.

Initially, we ran HPLC assays using hydroxylamine as our nucleophile; here, we are largely monitoring peptide cleavage analogous to our plate reader and enzyme kinetics assays. Indeed, we saw that the P94D saSrtA5M variant outperformed saSrtA5M for both the LPETG and LPKTG substrates at all time points in our assay, with *t* = 2 h substrate conversion values of 74.1 ± 3.8% (LPETG) and 79.1 ± 3.3% (LPKTG) in triplicate experiments (Figures 11B–C and S6A). On average, these values were approximately 20–25% better than saSrtA5M with the LPETG substrate (62.7 ± 1.7%, at *t* = 2 h) (Figures 11B–C and S6A). Like our fluorescence assay results, SML efficiency was dramatically reduced for saSrtA5M with the LPKTG peptide, with 28.7 ± 0.01% substrate conversion at *t* = 2 h (Figures 11B–C, S6A). Although saSrtA5M and P94D saSrtA5M outperformed the single P94D saSrtA mutation for the LPETG substrate, product conversion was very similar at all time points for P94D saSrtA + LPETG as compared to saSrtA5M + LPKTG. In our peptide cleavage assay, relative fluorescence units at *t* = 2 h were similar for all P2 amino acids (A, E, K, S). Here, however, the P94D saSrtA enzyme showed a stronger preference for the LPKTG sequence (40.6 ± 1.5%, at *t* = 2 h) over LPETG, with a 1.4-fold increase in substrate conversion (Figures 6, 11B–C, S6A). This P2 preference for Lys by P94D saSrtA was also apparent when we normalized *t* = 20 min RFU values to LPETG in our peptide cleavage assay (Figure 5A). Overall, the SML experiments using peptide substrates revealed a greater increase in product formation with P94D saSrtA5M as compared to the P94R-containing saSrtA5M enzyme, although including the pentamutant background (i.e., additional four mutations, D160N/D165A/K190E/K196T) still provided the greatest cleavage efficiency difference, in the presence of a mutation at P94, for the LPETG peptide.

### Sortase Mediated Ligation Reactions Using a Gly–Gly Nucleophile

Finally, we ran sortase-mediated ligation reactions using a standard glycine (Gly–Gly) nucleophile. As described in the Materials and Methods, we used HPLC to measure reaction progress (as substrate conversion %) over 2 h with either an LPETG or LPKTG substrate (full sequence: Abz-LPXTGGK­(Dnp), as in other peptide assays) and an excess of diglycine (GG) peptide nucleophile. Here, we chose to compare the saSrtA5M and P94D saSrtA5M variants to see if the P94D mutation affected SML efficiency (Figures 11D–S6A). Consistent with our other results, while we saw a similar degree of cleavage product (GGK­(Dnp)) formation at all time points for the LPETG substrate, there was a substantial difference between saSrtA5M (46 ± 5%) as compared to P94D saSrtA5M (87 ± 2%) at *t* = 2 h using the LPKTG substrate (Figures 11D and S6A). In order to directly assess the formation of the desired ligation products (Abz-LPXTGG), the reactions were also characterized by LC–MS. In all cases clear signals for the ligation products were observed, with little to no hydrolysis product (<0.5% relative to ligation products) as estimated by mass spectrometry (representative mass spectrum shown in Figure S6B). These assays confirm that for certain LPXTG sequences (e.g., LPKTG) variants of saSrtA5M (e.g., P94D saSrtA5M) substantially outperform the original P94R-containing saSrtA5M enzyme in sortase-mediated ligation experiments. These experiments also demonstrate that the P94D mutation does not appear to have deleterious effects on the ratio of ligation product versus undesired substrate hydrolysis.

## Conclusions

Sortase-mediated ligation (SML) is a powerful technique used in a variety of protein engineering approaches.^26^ Historically, challenges in the utilization of SML included reversibility of the reaction, off-target hydrolysis of substrate, and a relatively low catalytic efficiency of the sortase catalytic domain.^26,57^ Within the SML engineering field, the one enzyme that is most widely used is saSrtA; specifically, engineered variants including a calcium-independent version of the pentamutant referenced here, termed the heptamutant (saSrtA7M) as discussed above. We previously characterized a structurally conserved loop in class A sortases, the β7-β8 loop, with a focus on Streptococcus SrtA enzymes.^36,51,52^ Analyses of experimental structures of saSrtA revealed an interaction between P94 and Y187 (in the β7–β8 loop) that is only present in the apo enzyme and which is not conserved in other SrtA enzymes (Figure 1C). We wanted to better understand the role of this position in saSrtA activity and specificity, and were additionally intrigued as P94 is a mutated residue in the catalytically enhanced pentamutant saSrtA5M, which contains P94R.^44^

As previously observed, we predicted that mutation at P94 primarily affects the overall *K*_m_ of saSrtA. We found a wide range of relative activities for P94X variants and substrate peptides, with any mutation at this residue resulting in similar or better overall activity for all enzyme–substrate pairs tested (Figures 2, 4). In our data, the P94R variant did not emerge as one of the most active, and indeed, we hypothesize that the presence of the P2 Glu (LPETG) may have influenced the original directed evolution results. The LPETG sequence is derived from an endogenous target of saSrtA, the Spa protein, which may have evolved to optimize SrtA recognition and activity, as this was the most active sequence for almost all our variants, presumably due to the R197 amino acid in saSrtA.

This Arg residue (R197 in saSrtA) is highly conserved in sortase enzymes. Traditionally, it is considered one of the catalytic triad residues. Recent work from ourselves and others suggested that while important, R197 likely does not stabilize the oxyanion intermediate in a catalytic manner, but instead contributes to substrate recognition and binding.^26,32–34^ This is consistent with the data presented here and previously, wherein R197 likely stabilizes the substrate backbone, as well as may influence P2 specificity. Indeed, the same group that developed saSrt5M later used the technique to engineer a sortase variant, SrtAβ, for use as a diagnostic and therapeutic tool for neurodegenerative disease.^27^ SrtAβ preferentially recognizes the LMVGG sequence and contains a Tyr at position 94 and Ser at the R197 position.^27^ The data presented here is consistent with these results, in that mutation of R197S presumably reduces the positive charge in the substrate binding cleft, potentially increasing affinity for a P2 Val. While P94Y saSrtA behaved like WT in our assays, the hydrophobic nature of the LMVGG sequence may be better tolerated.

Overall, our data argues that a combination of rational design based on structure–function analyses and high throughput screening techniques may elucidate improved versions of SrtA for use in SML experiments. We were able to increase relative activity for certain substrates using pentamutant variants containing P94A and P94D mutations, and show that these variants (e.g., P94D saSrtA5M) can outperform saSrtA5M in SML experiments with certain LPXTG substrates (Figure 11C,D). Because previous work revealed that catalytic efficiency decreases in the heptamutant as compared to the pentamutant due to a 3-fold increase in *K*_m_, we did not test calcium-independent versions of our P94X variants here.^45,46^ We would expect a similar increase in relative *K*_m_ for any of the P94X variants tested; however, these effects provide additional support toward continued optimization of saSrtA variants for use in SML applications. Our data argues that a better understanding of the basic stereochemical mechanisms of SrtA activity and specificity can provide insight toward the design and development of next-generation tools in this wide-reaching field.

## Supplementary Material



## Notes

Table with mass spectrometry analyses of saSrtA variants and synthesized peptides used-Table with initial velocities calculated (in RFU/min) for all enzyme–substrate pairs tested Full 2 h fluorescence data for the activity assays in Figures 2, 4 Initial velocity curves and linear fits for P94X saSrtA variants Graphical comparison of initial velocities for P94X saSrtA variants Supporting Information for the AlphaFold3 models generated, including output quality graphs and structural alignments with experimentally determined structures Replicate data for enzyme kinetics assays in Figure 11 Representative HPLC traces for assays with saSrtA5M, P94D saSrtA5M, and P94D saSrtA, with H_2_NOH and/or Gly–Gly nucleophiles (PDF)

## References

[1] Rajagopal M., Walker S. (2015). Envelope Structures of Gram-Positive Bacteria. Curr. Top. Microbiol. Immunol..

[2] Raz A., Fischetti V. A. (2008). Sortase A Localizes to Distinct Foci on the Streptococcus
Pyogenes Membrane. Proc. Natl. Acad. Sci. U.S.A..

[3] Ton-That H., Liu G., Mazmanian S. K., Faull K. F., Schneewind O. (1999). Purification
and Characterization of Sortase, the Transpeptidase That Cleaves Surface
Proteins of *Staphylococcus Aureus* at the LPXTG Motif. Proc. Natl. Acad. Sci. U.S.A..

[4] Mazmanian S. K., Liu G., Ton-That H., Schneewind O. (1999). *Staphylococcus
Aureus* Sortase, an Enzyme That Anchors Surface Proteins to
the Cell Wall. Science.

[5] Ton-That H., Schneewind O. (1999). Anchor Structure of *Staphylococcal* Surface Proteins. IV. Inhibitors of the Cell Wall Sorting Reaction. J. Biol. Chem..

[6] Zrelovs N., Kurbatska V., Rudevica Z., Leonchiks A., Fridmanis D. (2021). Sorting out the Superbugs: Potential of Sortase A Inhibitors
among Other Antimicrobial Strategies to Tackle the Problem of Antibiotic
Resistance. Antibiotics (Basel).

[7] Jaudzems K., Kurbatska V., Je Kabsons A., Bobrovs R., Rudevica Z., Leonchiks A. (2020). Targeting
Bacterial Sortase A with Covalent Inhibitors:
27 New Starting Points for Structure-Based Hit-to-Lead Optimization. ACS Infect. Dis..

[8] Cascioferro S., Raffa D., Maggio B., Raimondi M. V., Schillaci D., Daidone G. (2015). Sortase A Inhibitors:
Recent Advances and Future Perspectives. J.
Med. Chem..

[9] Thappeta K. R. V., Zhao L. N., Nge C. E., Crasta S., Leong C. Y., Ng V., Kanagasundaram Y., Fan H., Ng S. B. (2020). In-Silico
Identified New Natural Sortase A Inhibitors Disrupt *S. Aureus* Biofilm Formation. Int. J. Mol. Sci..

[10] Chan A. H., Wereszczynski J., Amer B. R., Yi S. W., Jung M. E., McCammon J. A., Clubb R. T. (2013). Discovery of *Staphylococcus
Aureus* Sortase A Inhibitors Using Virtual Screening and the
Relaxed Complex Scheme. Chem. Biol. Drug Des..

[11] Nitulescu G., Zanfirescu A., Olaru O. T., Nicorescu I. M., Nitulescu G. M., Margina D. (2016). Structural Analysis of Sortase A
Inhibitors. Molecules.

[12] Maresso A. W., Wu R., Kern J. W., Zhang R., Janik D., Missiakas D. M., Duban M.-E., Joachimiak A., Schneewind O. (2007). Activation
of Inhibitors by Sortase Triggers Irreversible Modification of the
Active Site. J. Biol. Chem..

[13] Wójcik M., Eleftheriadis N., Zwinderman M. R. H., Dömling A. S. S., Dekker F. J., Boersma Y. L. (2019). Identification of Potential Antivirulence
Agents by Substitution-Oriented Screening for Inhibitors of *Streptococcus Pyogenes* Sortase A. Eur. J. Med. Chem..

[14] Gosschalk J. E., Chang C., Sue C. K., Siegel S. D., Wu C., Kattke M. D., Yi S. W., Damoiseaux R., Jung M. E., Ton-That H. (2020). A Cell-Based
Screen
in *Actinomyces Oris* to Identify Sortase Inhibitors. Sci. Rep..

[15] Zhang J., Liu H., Zhu K., Gong S., Dramsi S., Wang Y.-T., Li J., Chen F., Zhang R., Zhou L. (2014). Antiinfective
Therapy with a Small Molecule Inhibitor of *Staphylococcus
Aureus* Sortase. Proc. Natl. Acad. Sci.
U.S.A..

[16] Volynets G. P., Barthels F., Hammerschmidt S. J., Moshynets O. V., Lukashov S. S., Starosyla S. A., Vyshniakova H. V., Iungin O. S., Bdzhola V. G., Prykhod’ko A. O. (2022). Identification of Novel Small-Molecular Inhibitors of *Staphylococcus
Aureus* Sortase A Using Hybrid Virtual Screening. J. Antibiot..

[17] Sapra R., Rajora A. K., Kumar P., Maurya G. P., Pant N., Haridas V. (2021). Chemical Biology of
Sortase A Inhibition: A Gateway
to Anti-Infective Therapeutic Agents. J. Med.
Chem..

[18] Scott C. J., McDowell A., Martin S. L., Lynas J. F., Vandenbroeck K., Walker B. (2002). Irreversible Inhibition
of the Bacterial Cysteine Protease-Transpeptidase
Sortase (SrtA) by Substrate-Derived Affinity Labels. Biochem. J..

[19] Kim S.-W., Chang I.-M., Oh K.-B. (2002). Inhibition
of the Bacterial Surface
Protein Anchoring Transpeptidase Sortase by Medicinal Plants. Biosci. Biotechnol. Biochem..

[20] Obeng E. M., Fulcher A. J., Wagstaff K. M. (2023). Harnessing Sortase A Transpeptidation
for Advanced Targeted Therapeutics and Vaccine Engineering. Biotechnol. Adv..

[21] Braga
Emidio N., Cheloha R. W. (2024). Semi-Synthetic Nanobody-Ligand Conjugates
Exhibit Tunable Signaling Properties and Enhanced Transcriptional
Outputs at Neurokinin Receptor-1. Protein Sci..

[22] Zou Z., Ji Y., Schwaneberg U. (2023). Empowering Site-Specific Bioconjugations In Vitro and
In Vivo: Advances in Sortase Engineering and Sortase-Mediated Ligation. Angew. Chem., Int. Ed..

[23] Beerli R. R., Hell T., Merkel A. S., Grawunder U. (2015). Sortase Enzyme-Mediated
Generation of Site-Specifically Conjugated Antibody Drug Conjugates
with High In Vitro and In Vivo Potency. PLoS
One.

[24] Gébleux R., Briendl M., Grawunder U., Beerli R. R. (2019). Sortase A Enzyme-Mediated
Generation of Site-Specifically Conjugated Antibody-Drug Conjugates. Methods Mol. Biol..

[25] Kumari P., Bowmik S., Paul S. K., Biswas B., Banerjee S. K., Murty U. S., Ravichandiran V., Mohan U., Sortase A. (2021). A Chemoenzymatic
Approach for the Labeling of Cell Surfaces. Biotechnol. Bioeng..

[26] Amacher J.
F., Antos J. M. (2024). Sortases:
Structure, Mechanism, and Implications for
Protein Engineering. Trends Biochem. Sci..

[27] Podracky C. J., An C., DeSousa A., Dorr B. M., Walsh D. M., Liu D. R. (2021). Laboratory
Evolution of a Sortase Enzyme That Modifies Amyloid-β Protein. Nat. Chem. Biol..

[28] Park C., Zhang Y., Jung J. U., Buron L. D., Lin N.-P., Hoeg-Jensen T., Chou D. H.-C. (2023). Antagonistic Insulin Derivative Suppresses
Insulin-Induced Hypoglycemia. J. Med. Chem..

[29] Moliner-Morro A., J Sheward D., Karl V., Perez Vidakovics L., Murrell B., McInerney G. M., Hanke L. (2020). Picomolar SARS-CoV-2
Neutralization Using Multi-Arm PEG Nanobody Constructs. Biomolecules.

[30] Spirig T., Weiner E. M., Clubb R. T. (2011). Sortase
Enzymes in Gram-Positive
Bacteria. Mol. Microbiol..

[31] Jacobitz A. W., Kattke M. D., Wereszczynski J., Clubb R. T. (2017). Sortase Transpeptidases:
Structural Biology and Catalytic Mechanism. Adv. Protein Chem. Struct. Biol..

[32] Tian B.-X., Eriksson L. A. (2011). Catalytic Mechanism and Roles of
Arg197 and Thr183
in the *Staphylococcus Aureus* Sortase A Enzyme. J. Phys. Chem. B.

[33] Johnson D.
A., Piper I. M., Vogel B. A., Jackson S. N., Svendsen J. E., Kodama H. M., Lee D. E., Lindblom K. M., McCarty J., Antos J. M. (2022). Structures of *Streptococcus Pyogenes* Class A Sortase
in Complex with Substrate and Product Mimics Provide
Key Details of Target Recognition. J. Biol.
Chem..

[34] Chen J.-L., Wang X., Yang F., Li B., Otting G., Su X.-C. (2023). 3D Structure of the Transient Intermediate
of the Enzyme–substrate
Complex of Sortase A Reveals How Calcium Binding and Substrate Recognition
Cooperate in Substrate Activation. ACS Catal..

[35] Malik A., Kim S. B. (2019). A Comprehensive in Silico Analysis
of Sortase Superfamily. J. Microbiol..

[36] Valgardson J. D., Struyvenberg S. A., Sailer Z. R., Piper I. M., Svendsen J. E., Johnson D. A., Vogel B. A., Antos J. M., Harms M. J., Amacher J. F. (2022). Comparative
Analysis and Ancestral Sequence Reconstruction
of Bacterial Sortase Family Proteins Generates Functional Ancestral
Mutants with Different Sequence Specificities. Bacteria.

[37] Nikghalb K. D., Horvath N. M., Prelesnik J. L., Banks O. G. B., Filipov P. A., Row R. D., Roark T. J., Antos J. M. (2018). Expanding the Scope
of Sortase-Mediated Ligations by Using Sortase Homologues. ChemBioChem.

[38] Schmohl L., Bierlmeier J., von Kügelgen N., Kurz L., Reis P., Barthels F., Mach P., Schutkowski M., Freund C., Schwarzer D. (2017). Identification
of Sortase Substrates
by Specificity Profiling. Bioorg. Med. Chem..

[39] Ilangovan U., Ton-That H., Iwahara J., Schneewind O., Clubb R. T. (2001). Structure of Sortase, the Transpeptidase
That Anchors
Proteins to the Cell Wall of *Staphylococcus Aureus*. Proc. Natl. Acad. Sci. U.S.A..

[40] Suree N., Liew C. K., Villareal V. A., Thieu W., Fadeev E. A., Clemens J. J., Jung M. E., Clubb R. T. (2009). The Structure of
the *Staphylococcus Aureus* Sortase-Substrate Complex
Reveals How the Universally Conserved LPXTG Sorting Signal Is Recognized. J. Biol. Chem..

[41] Chan A. H., Yi S. W., Terwilliger A. L., Maresso A. W., Jung M. E., Clubb R. T. (2015). Structure of the *Bacillus Anthracis* Sortase A Enzyme Bound to Its Sorting Signal: A FLEXIBLE AMINO-TERMINAL
APPENDAGE MODULATES SUBSTRATE ACCESS. J. Biol.
Chem..

[42] Weiner E. M., Robson S., Marohn M., Clubb R. T. (2010). The Sortase A Enzyme
That Attaches Proteins to the Cell Wall of *Bacillus Anthracis* Contains an Unusual Active Site Architecture. J. Biol. Chem..

[43] Race P. R., Bentley M. L., Melvin J. A., Crow A., Hughes R. K., Smith W. D., Sessions R. B., Kehoe M. A., McCafferty D. G., Banfield M. J. (2009). Crystal Structure
of *Streptococcus Pyogenes* Sortase A: Implications
for Sortase Mechanism. J. Biol. Chem..

[44] Chen I., Dorr B. M., Liu D. R. (2011). A General Strategy
for the Evolution
of Bond-Forming Enzymes Using Yeast Display. Proc. Natl. Acad. Sci. U.S.A..

[45] Hirakawa H., Ishikawa S., Nagamune T. (2012). Design of Ca2+-Independent *Staphylococcus Aureus* Sortase A Mutants. Biotechnol. Bioeng..

[46] Hirakawa H., Ishikawa S., Nagamune T. (2015). Ca2+ -Independent
Sortase-A Exhibits
High Selective Protein Ligation Activity in the Cytoplasm of *Escherichia Coli*. Biotechnol. J..

[47] Witte M. D., Wu T., Guimaraes C. P., Theile C. S., Blom A. E. M., Ingram J. R., Li Z., Kundrat L., Goldberg S. D., Ploegh H. L. (2015). Site-Specific Protein
Modification Using Immobilized Sortase in Batch and Continuous-Flow
Systems. Nat. Protoc..

[48] Bentley M. L., Gaweska H., Kielec J. M., McCafferty D. G. (2007). Engineering
the Substrate Specificity of *Staphylococcus Aureus* Sortase A. The Beta6/Beta7 Loop from SrtB Confers NPQTN Recognition
to SrtA. J. Biol. Chem..

[49] Ugur I., Schatte M., Marion A., Glaser M., Boenitz-Dulat M., Antes I. (2018). Ca2+ Binding Induced Sequential Allosteric Activation of Sortase
A: An Example for Ion-Triggered Conformational Selection. PLoS One.

[50] Piotukh K., Geltinger B., Heinrich N., Gerth F., Beyermann M., Freund C., Schwarzer D. (2011). Directed Evolution of Sortase A Mutants
with Altered Substrate Selectivity Profiles. J. Am. Chem. Soc..

[51] Piper I. M., Struyvenberg S. A., Valgardson J. D., Johnson D. A., Gao M., Johnston K., Svendsen J. E., Kodama H. M., Hvorecny K. L., Antos J. M. (2021). Sequence Variation in the Β7-Β8
Loop of Bacterial Class A Sortase Enzymes Alters Substrate Selectivity. J. Biol. Chem..

[52] Gao M., Johnson D. A., Piper I. M., Kodama H. M., Svendsen J. E., Tahti E., Longshore-Neate F., Vogel B., Antos J. M., Amacher J. F. (2022). Structural and Biochemical
Analyses of Selectivity
Determinants in Chimeric *Streptococcus* Class A Sortase
Enzymes. Protein Sci..

[53] Kodama H. M., Lindblom K. M., Walkenhauer E. G., Antos J. M., Amacher J. F. (2024). Amino Acid
Variability at W194 of *Staphylococcus Aureus* Sortase
A Alters Nucleophile Specificity. Protein Sci..

[54] Abramson J., Adler J., Dunger J., Evans R., Green T., Pritzel A., Ronneberger O., Willmore L., Ballard A. J., Bambrick J. (2024). Accurate Structure Prediction of Biomolecular
Interactions with AlphaFold 3. Nature.

[55] Gao X., Dong X., Li X., Liu Z., Liu H. (2020). Prediction
of Disulfide Bond Engineering Sites Using a Machine Learning Method. Sci. Rep..

[56] Vogel B. A., Blount J. M., Kodama H. M., Goodwin-Rice N. J., Andaluz D. J., Jackson S. N., Antos J. M., Amacher J. F. (2024). A Unique
Binding Mode of P1’ Leu-Containing Target Sequences for *Streptococcus Pyogenes* Sortase A Results in Alternative
Cleavage. RSC Chem. Biol..

[57] Morgan H. E., Turnbull W. B., Webb M. E. (2022). Challenges in the Use of Sortase
and Other Peptide Ligases for Site-Specific Protein Modification. Chem. Soc. Rev..

[58] Frankel B.
A., Kruger R. G., Robinson D. E., Kelleher N. L., McCafferty D. G. (2005). *Staphylococcus Aureus* Sortase Transpeptidase SrtA: Insight
into the Kinetic Mechanism and Evidence for a Reverse Protonation
Catalytic Mechanism. Biochemistry.

[59] Huang X., Aulabaugh A., Ding W., Kapoor B., Alksne L., Tabei K., Ellestad G. (2003). Kinetic Mechanism of *Staphylococcus
Aureus* Sortase SrtA. Biochemistry.

[60] Wang X., Chen J.-L., Otting G., Su X.-C. (2018). Conversion of an
Amide to a High-Energy Thioester by *Staphylococcus Aureus* Sortase A Is Powered by Variable Binding Affinity for Calcium. Sci. Rep..

[61] Kruger R. G., Otvos B., Frankel B. A., Bentley M., Dostal P., McCafferty D. G. (2004). Analysis
of the Substrate Specificity of the *Staphylococcus Aureus* Sortase Transpeptidase SrtA. Biochemistry.

[62] Biswas T., Pawale V. S., Choudhury D., Roy R. P. (2014). Sorting of LPXTG
Peptides by Archetypal Sortase A: Role of Invariant Substrate Residues
in Modulating the Enzyme Dynamics and Conformational Signature of
a Productive Substrate. Biochemistry.

[63] Park, J. ; Lee, E. H. ; Song, Y. H. ; Cho, S. ; Jang, Y. ; Choi, S. ; Kim, D. ; Heo, Y. Structural Insights into the Enhanced Catalytic Efficiency of the Engineered Sortase A Pentamutant. Bull. Korean Chem. Soc. 2026.

[64] Wang C., Desmet R., Snella B., Vicogne J., Melnyk O., Agouridas V. (2025). Leveraging Sortase A Electrostatics for Powerful Transpeptidation
Reactions. Angew. Chem., Int. Ed..

[65] Chen L., Cohen J., Song X., Zhao A., Ye Z., Feulner C. J., Doonan P., Somers W., Lin L., Chen P. R. (2016). Improved Variants of SrtA for Site-Specific Conjugation
on Antibodies and Proteins with High Efficiency. Sci. Rep..

[66] Bentley M. L., Lamb E. C., McCafferty D. G. (2008). Mutagenesis Studies of Substrate
Recognition and Catalysis in the Sortase A Transpeptidase from *Staphylococcus Aureus*. J. Biol. Chem..

