# Supplementary material for: Amino Acid Variants at the P94 Position in Staphylococcus aureus Class a Sortase Modulate Substrate Binding and Enzyme Activity
Source: Biochemistry. 2026 Apr 2;65(8):1325–39. doi: 10.1021/acs.biochem.6c00099 (PMC13104033; doi:10.1021/acs.biochem.6c00099)
Supplement: Supplementary file 1 [file bi6c00099_si_001.pdf]

Supporting Information for

Amino acid variants at the P94 position in *Staphylococcus aureus* class A sortase modulate substrate binding and enzyme activity

Noah Cox-Tigre,[1] Mia E. Stewart,[1] Jackson Tucker,[1] Erich G. Walkenhauer,[1] Cooper S. Wilce,[1] John M. Antos,[1,*] and Jeanine F. Amacher[1,*]

[1]Department of Chemistry, Western Washington University, Bellingham, WA, USA, 98225-9008

*Corresponding Authors:

Jeanine Amacher, Department of Chemistry, Western Washington University, 516 High St – MS9150, Bellingham, WA, 98225, Tel: +1-360-650-4397, Fax: +1-360-650-2826
Email: amachej@wwu.edu

John Antos, Department of Chemistry, Western Washington University, 516 High St – MS9150, Bellingham, WA, 98225, Tel: +1-360-650-2271, Fax: +1-360-650-2826
Email: antosj@wwu.edu

Table of Contents

Table S1. Mass spectrometry analyses of saSrtA variants and synthesized peptides. S2

Table S2. Initial velocities (in RFU/min) for all enzymes/substrates tested. S3

Figure S1. Fluorescence data for saSrtA variants with LPXTG substrates (t=2 h). S4

Figure S2. Initial velocity calculations for P94X saSrtA variants. S5

Figure S3. Comparison of initial velocities for P94X saSrtA variants. S6

Figure S4. AlphaFold3 models of WT, P94D, and P94R saSrtA wth substrate and Ca^{2+}. S7

Figure S5. Replicate data for enzyme kinetics assays. S8

Figure S6. Representative HPLC traces for assays with saSrtA5M, P94D saSrtA5M, and P94D saSrtA (H_2NOH and/or Gly-Gly nucleophiles). S9

Table S1. Mass spectrometry analysis of all enzyme variants and peptide substrates / peptide reaction products. Predicted masses for enzyme variants were calculated using ExPasy ProtParam. Predicted and observed masses for protein variants represent average molecular weight (MW). Predicted and observed masses for peptides represent $[M+H]^+$ ions (monoisotopic). All peptide substrates (Abz-LP**X**TGGK(Dnp)) were synthesized with a C-terminal primary amide ($-NH_2$). Peptide ligation products (Abz-LP**X**TGG) contain a C-terminal carboxylic acid (-COOH).

**P94X**	**Predicted Mass (Da)**	**Observed Mass (Da)**
A	18710.0	18711.7
D	18754.0	18756.2
E	18768.1	18770.7
F	18786.1	18789.5
G	18696.0	18699.6
H	18776.1	18779.5
I	18752.1	18756.3
K	18767.1	18768.8
L	18752.1	18757.9
M	18770.1	18773.7
N	18753.1	18757.8
WT (P)	18736.1	18738.7
Q	18767.1	18771.4
R	18795.1	18799.6
S	18726.0	18730.3
T	18740.1	18739.7
V	18738.1	18741.4
W	18825.2	18829.1
Y	18802.1	18805.3
saSrtA5M	18724.0	18721.7
P94A 5M	18638.9	18636.7
P94D 5M	18682.9	18681.9
**Peptides**		
Abz-LP**A**TGGK(Dnp)	927.4	927.5
Abz-LP**E**TGGK(Dnp)	985.4	985.3
Abz-LP**K**TGGK(Dnp)	984.5	984.6
Abz-LP**S**TGGK(Dnp)	943.4	943.6
GGK(Dnp)	426.2	426.1
Abz-LP**E**TGG	692.3	692.3
Abz-LP**K**TGG	691.4	691.4

Table S2. Initial velocities (in RFU/min) for all enzymes/substrates tested. Data used for calculations is shown in **Figure S2**. Linear regression curves were calculated using GraphPad Prism. All R^2 values were ≥ 0.996, with most ≥ 0.999.

Substrate	LPATG		LPETG		LPKTG		LPSTG	
	Initial Velocity	Standard Deviation	Initial Velocity	Standard Deviation	Initial Velocity	Standard Deviation	Initial Velocity	Standard Deviation
**P94A**	2834.3	77.1	3760.0	65.6	1788.0	159.8	2749.0	109.3
**P94D**	3671.0	208.4	2968.3	105.7	3609.7	40.0	3803.0	110.8
**P94E**	2943.7	6.1	2469.3	161.6	3107.3	152.6	2930.3	116.6
**P94F**	786.2	20.3	871.7	63.6	480.5	34.3	662.5	26.0
**P94G**	3284.7	88.3	4310.0	206.9	2405.0	82.6	2972.3	229.6
**P94H**	1922.0	123.8	4012.7	173.3	1334.7	21.6	2172.0	60.2
**P94I**	1359.7	21.4	1592.3	58.0	877.5	17.4	1139.0	34.6
**P94K**	1168.7	34.8	2752.3	142.3	606.0	50.4	1090.0	20.7
**P94L**	1070.5	98.9	1546.3	16.3	783.0	45.9	1140.7	71.8
**P94M**	1098.7	50.8	1364.3	27.2	845.2	50.8	1036.3	24.6
**P94N**	1826.0	47.7	2865.0	61.3	1349.3	5.1	2172.0	77.7
**WT (P94)**	598.2	24.8	937.8	47.6	467.2	14.8	610.0	6.3
**P94Q**	1726.0	61.0	2971.3	250.8	1397.7	15.3	1717.7	37.6
**P94R**	722. 7	25.5	2140.0	49.8	405.2	34.9	901.3	43.8
**P94S**	2764.3	27.6	4214.7	133.1	2326.7	54.5	3158.3	148.0
**P94T**	3428.3	37.6	4788.0	125.0	2909.0	64.1	3568.7	95.3
**P94V**	1172.0	56.3	1636.3	97.1	950.3	34.6	1187.2	179.3
**P94W**	875.2	31.2	1400.3	68.5	624.5	30.8	716.3	29.8
**P94Y**	987.3	48.3	1320.0	60.1	630.7	0.8	779.8	18.2
**saSrtA5M**	520.7	50.9	1285.3	58.6	339.7	15.0	617.5	21.8
**P94A 5M**	730.8	24.8	1472.0	34.9	711.7	18.7	859.2	59.5
**P94D 5M**	846.3	12.3	1128.0	52.0	1107.0	22.3	931.2	99.3

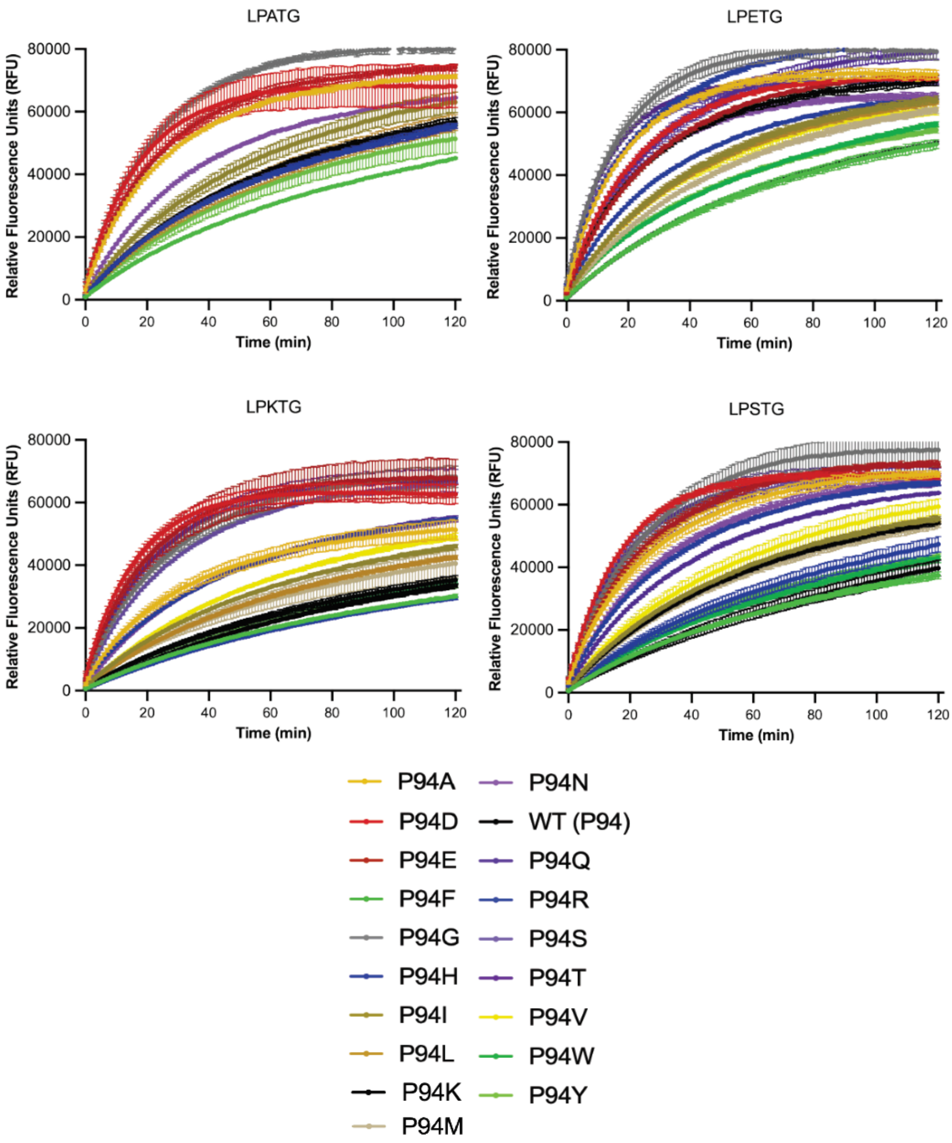

Figure S1. Fluorescence data for saSrtA variants with LPXTG substrates (t=2 h). The entire time course (t=2 h) for the activity assays is shown. This data matches that shown for t=60 min in **Figures 2A and 4**. Data is shown as the average of triplicate assays, with error bars equal to the standard deviation for each time point.

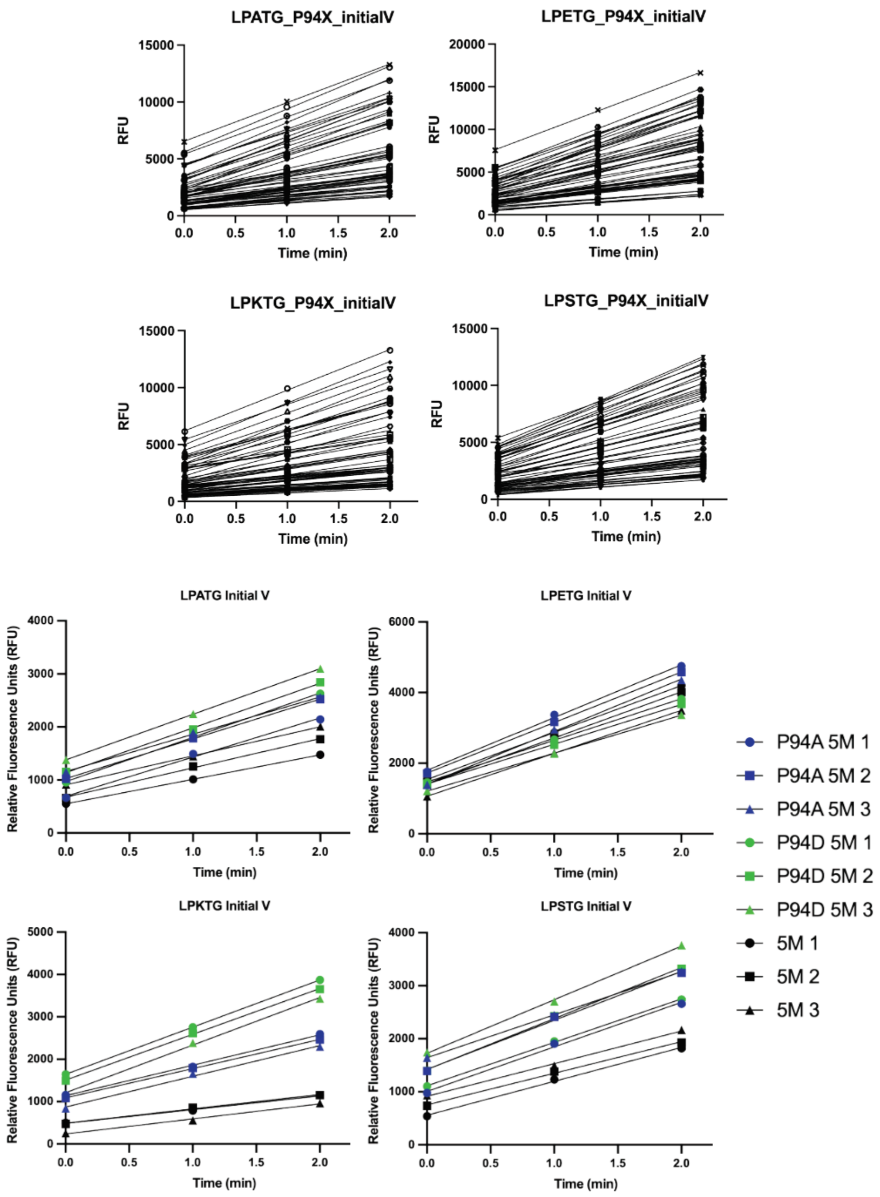

Figure S2. Initial velocity calculations for P94X saSrtA variants. The first 3 time points from fluorescence cleavage assays were used to calculate initial velocities for all enzyme-substrate pairs reported. Averaged values ± standard deviations are reported in **Table S2**.

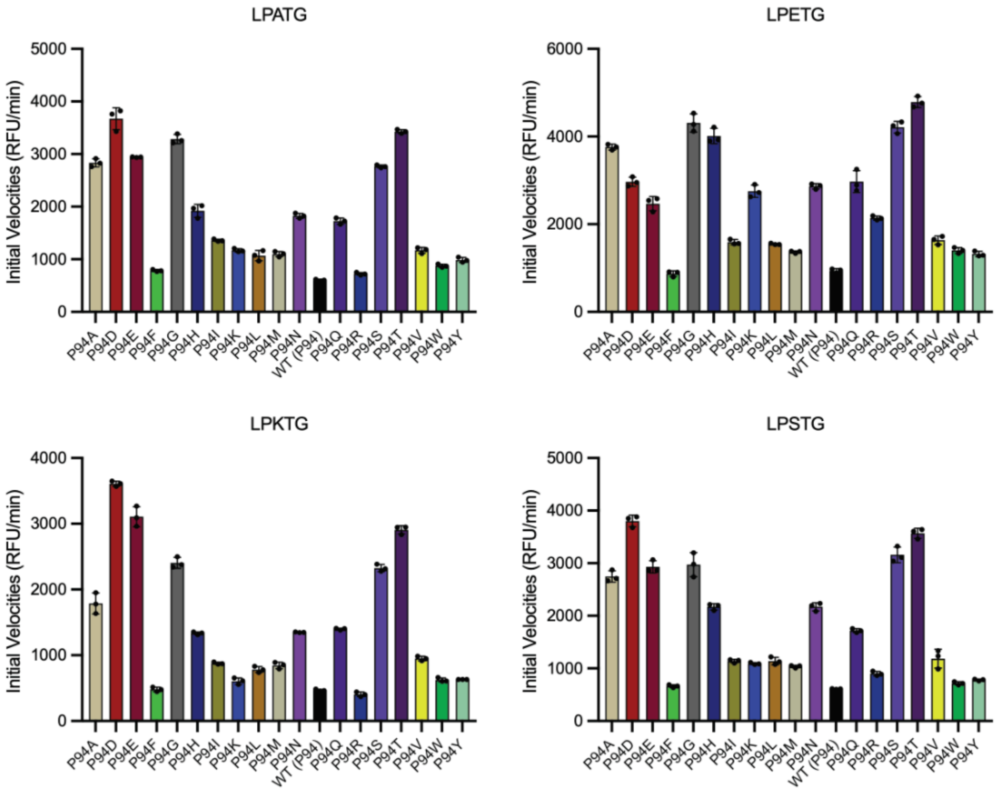

Figure S3. Comparison of initial velocities for P94X saSrtA variants. Initial velocities (RFU/min) are shown as the averaged values from triplicate assays ± standard deviation for four peptide substrates: LP**A**TG, LP**E**TG, LP**K**TG, and LP**S**TG. The trends observed are very similar to those for the RFU values at t=20 min (**Figures 2B, 4**).

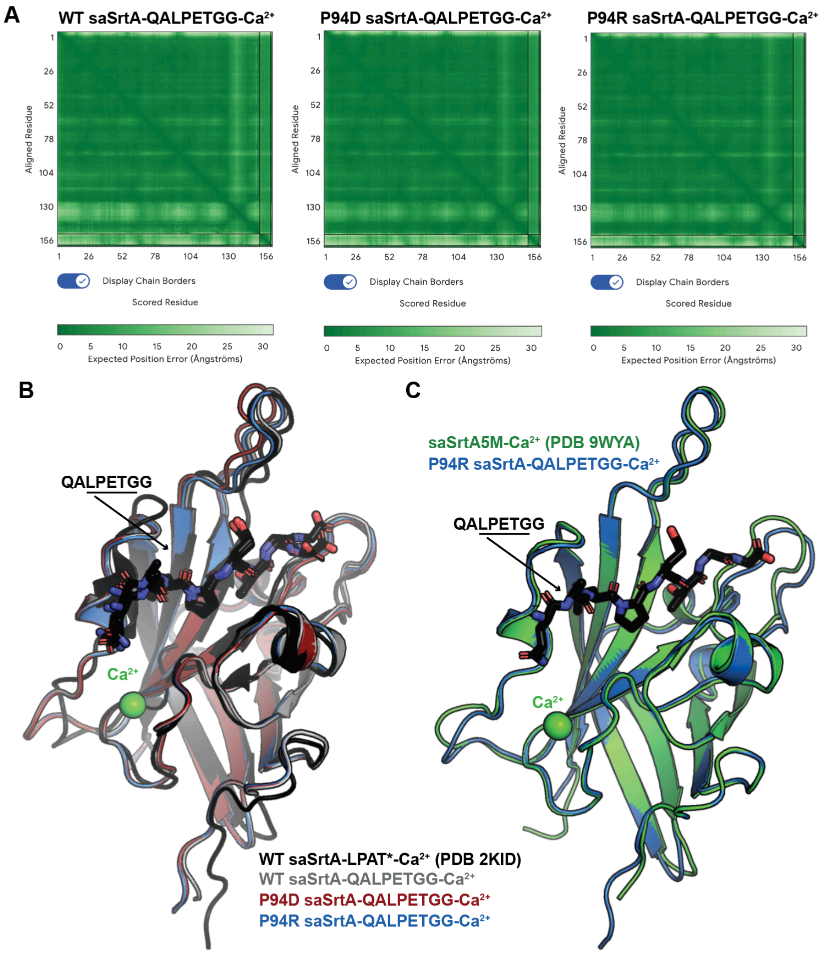

Figure S4. AlphaFold3 models of WT, P94D, and P94R saSrtA wth substrate and Ca^{2+}. (**A**) Output AlphaFold3 data to highlight the quality of the models analyzed. The corresponding output values (pTM, ipTM) are: for WT saSrtA-QALPETGG-Ca^{2+}, ipTM = 0.62, pTM = 0.87; P94D saSrtA-QALPETGG-Ca^{2+}, ipTM = 0.57, pTM = 0.88; and P94R saSrtA-QALPETGG-Ca^{2+}, ipTM = 0.6, pTM = 0.89. (**B**) All models are shown in cartoon representation and colored by P94X saSrtA variant, as labeled. The QALPETGG peptides are shown as sticks and colored by heteroatom (C=black, N=blue, O=red). The Ca^{2+} ion is a green sphere and labeled. Although only the model_0 is shown for each, all output models aligned with RMSD < 0.2 Å. The models are also compared to the NMR structure of saSrtA-LPAT* (PDB ID 2KID). The WT saSrtA-QALPETGG-Ca^{2+} model and 2KID structure were aligned with RMSD = 1.228 Å (over 1015 atoms). (**C**) Alignment of the P94R saSrtA-QALPETGG-Ca^{2+} model with saSrtA5M + Ca^{2+} (PDB ID 9WYA), RMSD = 0.360 Å (over 842 atoms). Structures are shown as in (**B**) and colored as labeled.

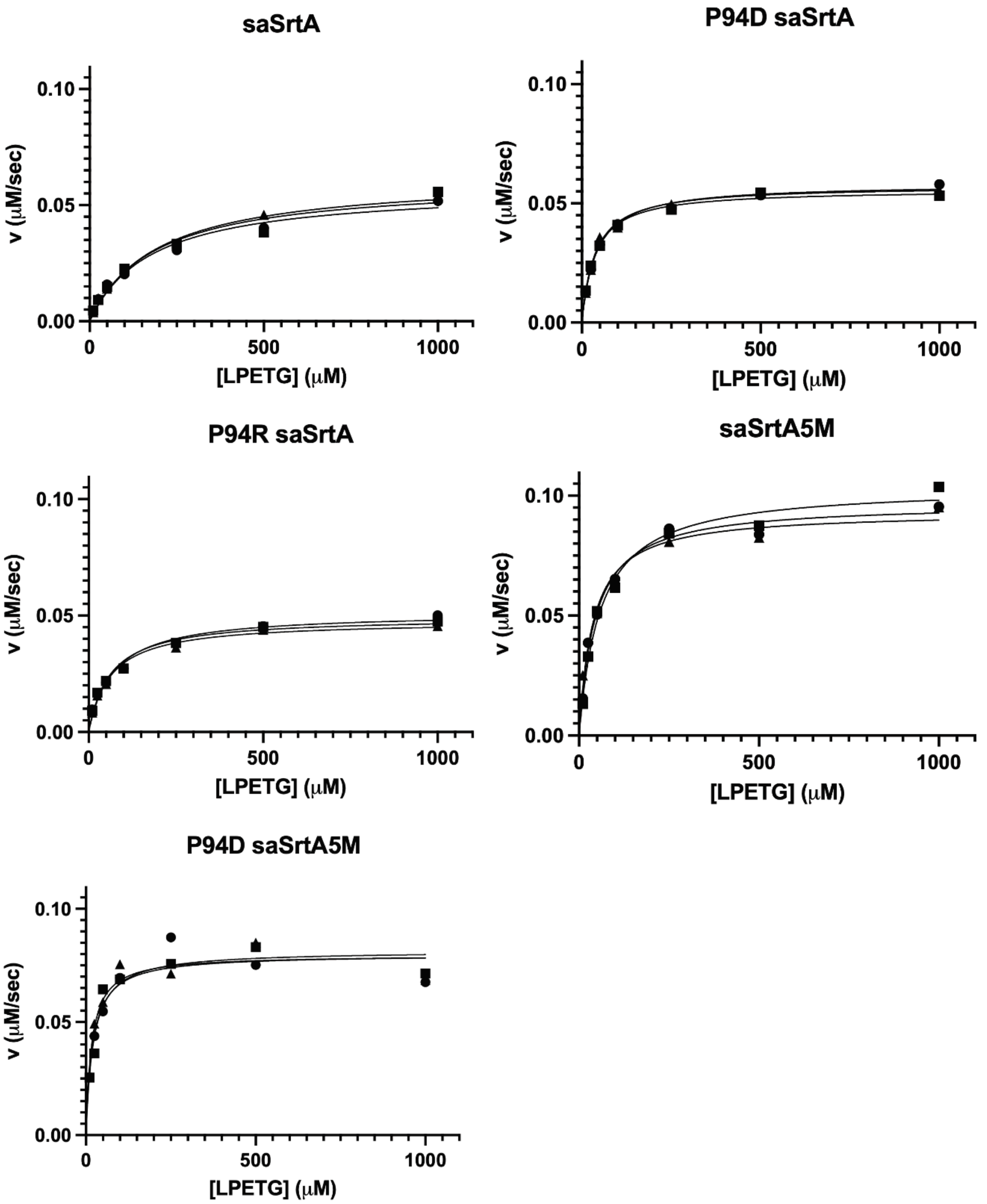

Figure S5. Replicate data for enzyme kinetics assays. Replicate data for enzyme kinetics assays (averaged data in **Figure 11A**). Reaction rates were calculated by determining the percentage of product formed at t=10, 60, 120, and 180 s using an HPLC assay. Rates were then determined using a linear regression curve in Excel. All R^2 values were >0.98. Parameters (V_{max} and K_m) were determined using the Michaelis-Menten fit in GraphPad Prism. For k_{cat}, this value was determined using the equation $V_{max} = k_{cat}$*[Enzyme]$_{total.}$

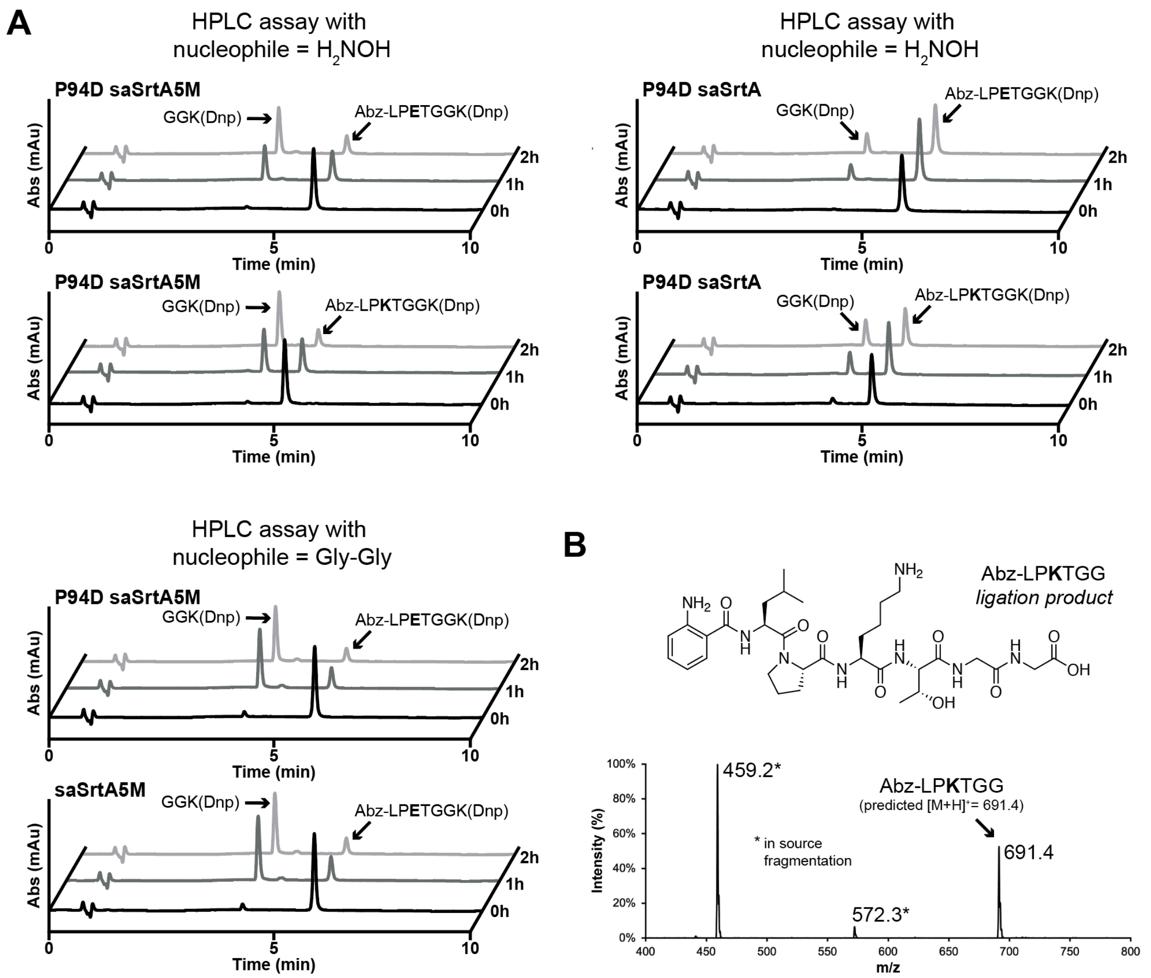

Figure S6. Representative HPLC traces (360 nm) for assays with saSrtA5M, P94D saSrtA5M, and P94D saSrtA (H_2NOH and/or Gly-Gly nucleophiles). (**A**) Representative HPLC traces of sortase-mediated ligations (H_2NOH or Gly-Gly nucleophiles) that show product formation over time, which can be calculated by integration of the indicated peaks (Abz-LP**X**TGGK(Dnp) and GGK(Dnp)). (**B**) Representative mass spectrum for the ligation product (Abz-LP**K**TGG) formed from the reaction of Abz-LP**K**TGGK(Dnp) and Gly-Gly in the presence of P94D SrtA5M, including the molecular structure of the product.
